# Characterizing Student Reasoning with Multiple Representations of Molecular Structure

**DOI:** 10.1021/jacsau.6c00898

**Published:** 2026-08-05

**Authors:** Roshni G. Bhaskar, Austin Cook, Fridah Rotich, Sara Arévalo, Maia Popova

**Affiliations:** † Department of Chemistry and Biochemistry, University of North Carolina at Greensboro, Greensboro, North Carolina 27412, United States; ‡ Center for Instructional Excellence, Purdue University, West Lafayette, Indiana 47907, United States; § CNC School of Medicine, University of North Carolina at Chapel Hill, Chapel Hill, North Carolina 27514, United States

**Keywords:** representational competence, student reasoning, scientific argumentation, affordances and limitations, heuristics, multiple external representations, chemistry education research

## Abstract

Organic chemistry relies on a range of symbolic representations to convey molecular structure and behavior. While each representation has specific advantages, the multitude of representations introduced is a well-documented obstacle for many students, furthered by a lack of instructional support in using the appropriate representation(s) for a given context. The contextual use of representations requires knowledge of their affordances and limitations, which is a key area of student representational reasoning that we sought to investigate. Through video-based, semistructured interviews that incorporated student annotations, we explored how 28 organic chemistry students perceive and reason about the affordances and limitations of six commonly used molecular representations: condensed structures, Lewis structures, skeletal structures, wedge–dash diagrams, Newman projections, and chair conformations. We found that most students could identify affordances but struggled to recognize limitations, with proficiency varying widely across participants. Students often relied on heuristics-based reasoning, which was productive when grounded in structural evidence and multicomponent reasoning. Those demonstrating stronger reasoning made more accurate claims, identified affordances across more conceptual categories, and articulated more relevant affordances and limitations. These findings highlight the tight interconnectedness of reasoning, conceptual understanding, and representational competence and underscore the need for instructional interventions that explicitly scaffold representational reasoning.

## Graphical abstract

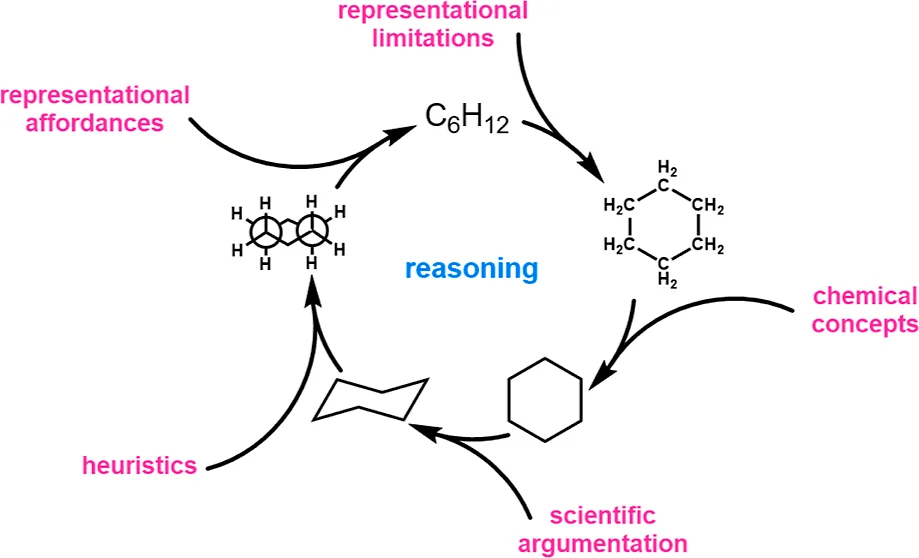

## Introduction

### Reasoning with Representations in Organic Chemistry

Organic chemistry, and chemistry more broadly, relies heavily on visual representations to infer causal relationships between chemical phenomena we observe in the macroscopic world and the underlying, invisible molecular interactions at the submicroscopic level.^1,2^ These representations include both internal mental models and external depictions such as diagrams, graphs, drawings, and chemical symbols, which are used to describe, explain, and predict chemical systems and behaviors.^3,4^ The use of visual representations is closely tied to how we reason in science. Science philosopher Jacob Bronowski pointed out that many mistakenly view reasoning and mental imagery as separate activities, when, in fact, “reasoning is constructed with movable images, just as certainly as poetry is.”^5^ The field of chemistry education research has increasingly explored students’ use of visual representations and the processes that accompany them;^6–8^ however, there remains limited consensus on how to integrate, operationalize, and scaffold student reasoning with representations in instructional contexts. In one such exploration, Talanquer calls for a more intentional, explicit, and granular approach to unpacking student reasoning with representations, careful consideration of the types of representational tasks that students engage in, and a focus on the role that heuristics play on student reasoning with representations.^8^

From the origins of organic chemistry through the present day, organic chemists have generated representations by “combining their mental images with complex chains of inference, from the sensual world right down to the microworld, using the heuristic symbolic tools of paper and wood.”^9^ From August Kekulé’s daydream by the fireplace (an internal visual representation) that inspired his theories of aromatic structure to van’t Hoff’s cardboard tetrahedrons (an external visual representation) that he sent to his European colleagues to aid their understanding of stereochemical theory,^9^ chemists have always used representation to generate ideas, test theories, and elaborate knowledge.^10^ Organic chemistry’s necessity to communicate abstract submicroscopic concepts has resulted in the development of a wide range of symbolic representations that encapsulate complex chemical information. To make sense of these representations, learners must unpack the encoded meaning by building connections, interpreting patterns, and generating new representations. Doing so requires familiarity with disciplinary conventions, classification systems, and/or procedural rules. Talanquer describes the process of unpacking a chemical representation as quite challenging as the process involves recognizing relevant explicit or implicit cues to activate and integrate multiple pieces of conceptual knowledge.^8^

Further, the field has evolved multiple ways of representing the same organic molecule, each with specific advantages (or affordances) and drawbacks (or limitations) in communicating chemical information. Identifying such affordances and limitations is among the representational competence skills described by Kozma and Russell.^11^

Selecting the appropriate visual representation for a particular chemical context is a related representational competence skill that we consider to be analogous to picking the appropriate tool for a specific carpentry project (Figure 1). This skill is intricately linked to the knowledge of representational affordances and limitations. Developing such an understanding, however, is challenging because students are often expected to learn both chemical concepts and the representations used to communicate those concepts simultaneously.

**Figure 1:**
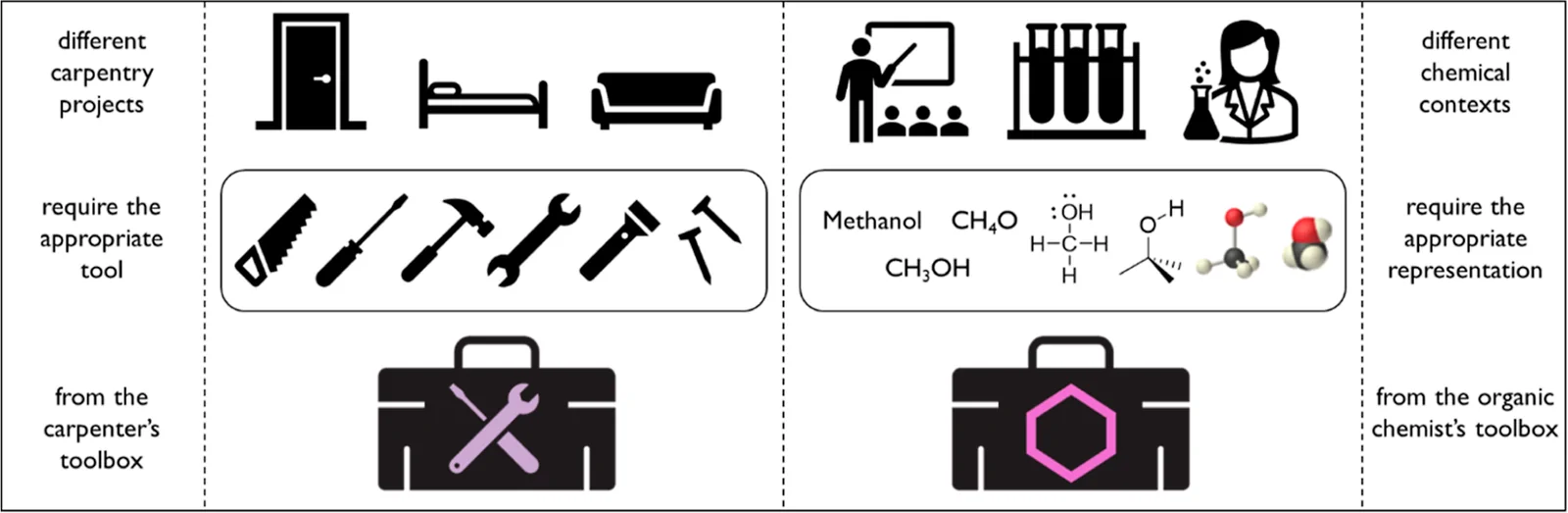
Toolbox analogy of representations in organic chemistry—just as a carpenter selects the right tool from their toolbox based on the needs of a specific project, a chemist chooses the most appropriate representation(s) for a given chemical context by considering the affordances and limitations of each.

This challenge encapsulates the representational dilemma,^12^ which refers to the cognitive challenge students face when they are expected to learn concepts and representations simultaneously without having fully mastered either. This dilemma represents a “chicken and egg problem,” where students need to understand the representation to make sense of the content, but they also need to understand the content to make sense of the representation.^4,13,14^ Not surprisingly, the literature shows that students struggle with (a) evaluating the purpose of different representations, (b) interpreting them, (c) translating between them, and (d) generating contextually appropriate representations on assessments.^7,15^ Additionally, the sheer number of representations introduced is a recurring, well-documented obstacle for many students, exacerbated by a lack of intentional instructional support in using representations.^11,16,17^

### Representational Competence

Representational competence, as described by Kozma and Russell, encompasses “a set of skills and practices that allow a person to reflectively use a variety of representations or visualizations, singly or together, to think about, communicate, and act on chemical phenomena in terms of underlying, aperceptual physical entities and processes.”^11^ In other words, these skills allow a learner to derive and communicate chemical meaning from representations (Figure 2). Underdeveloped and unsupported representational competence results in the use of primarily surface features and/or the algorithmic application of symbolic rules, while intentionally developed representational competence allows the purposeful use and generation of representations to make predictions, explain phenomena, support claims, and solve problems.^11^

**Figure 2:**
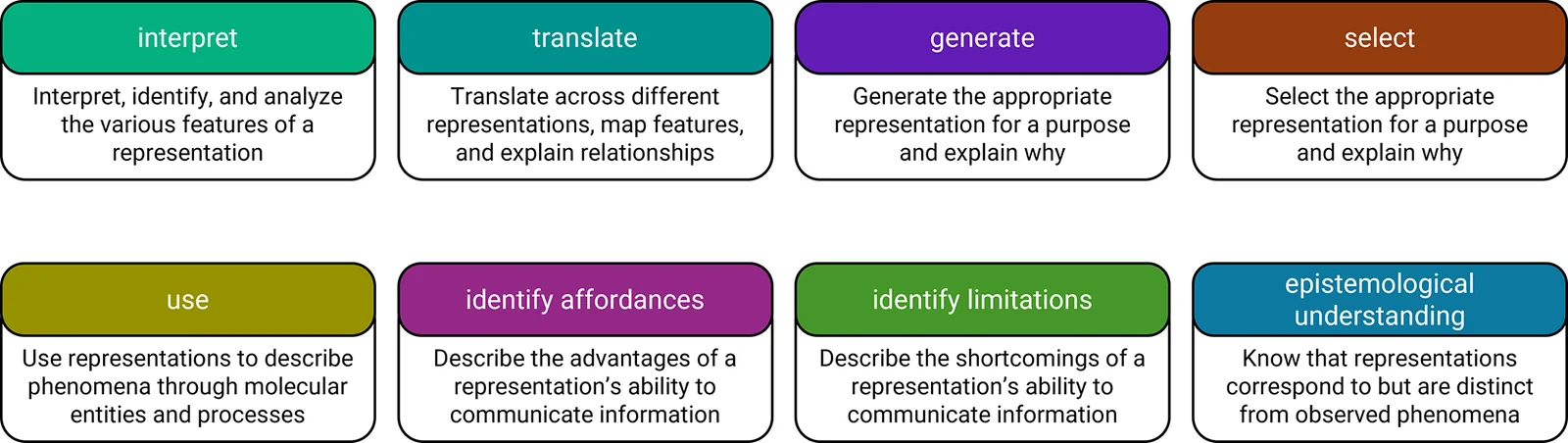
Representational competence skills.

To teach effectively about and with representations, educators must intentionally develop student representational competence, especially in visualizing and communicating relevant chemical phenomena. Educators must also realize the tight interconnectedness between various representational competence skills.^18^ For example, while the chemical interpretation of representations informs representations’ relative affordances and limitations, knowing the relative affordances and limitations of different chemical representations often informs the generation of, the use of, and the translation between representations. This places the ability to describe the affordances and limitations of multiple chemical representations at the nexus of the various representational competence skills.

This idea of representational affordances further draws from Gibson, who defines affordances as perceivable properties of an entity that are available to an observer.^19^ Furthermore, Greeno introduced the concept of representational constraints, which suggests that representations are defined not only by what they make visible and support interpretation of (affordances) but also by what they obscure or cannot readily communicate (constraints).^20,21^ Because our study focuses on students’ perceptions of what representations communicate and do not communicate, we use the term limitations broadly throughout the manuscript while recognizing its conceptual overlap with representational constraints.

The ability to describe the affordances and limitations is also linked to the idea of metarepresentational competence. Metarepresentational competence allows for the reflective and purposeful use of representations, which connects the “how” of working with representations to the deeper questions of “why” and “when” we use representations.^22,23^ While an understanding of representational affordances and limitations can support interpretation and reasoning about chemical structures (representational competence), a more sophisticated understanding also involves juxtaposing the epistemic value of various representations to reflect on their relative strengths, limitations, and contextual usefulness (metarepresentational competence).

### Evaluation of Instructional Resources to Support Student Proficiency across Multiple Representations

Several studies have found that representational competence remains unsupported in postsecondary chemistry classrooms. As described by diSessa, few widespread learning strategies are explicitly targeted at developing metarepresentational competence.^23^ This is corroborated by a textbook analysis of five commonly used organic chemistry textbooks that identified minimal support across all textbooks in explaining the affordances and limitations of different representations.^16^ When representations’ relative affordances and limitations are described, this is done very briefly through text, with no scaffolding or testing of this skill in worked examples or practice problems. Further, none of the textbooks cultivate the ability to select an appropriate representation for a particular purpose, suggesting that organic chemistry textbooks may fail to support learners in developing metarepresentational skills.^16^ Dávila and Talanquer found that general chemistry textbooks only allocate 1% of their items to translating between different levels of representation (i.e., from particulate to symbolic and vice versa) and identified a comparatively small number of questions requiring the interpretation or generation of graphical and symbolic forms of information.^24^ In interviews conducted with chemistry instructors, very few reported explicitly teaching and/or assessing students’ ability to explain the affordances and limitations of representations. Further, none reported teaching the ability to select the most appropriate representation for a particular purpose, once again highlighting a lack of focus on developing student metarepresentational competence.^17^ In a follow-up case study involving three organic chemistry instructors, it was found that none included activities that support students’ ability to select the appropriate representation for a particular purpose and the ability to explain the affordances and limitations of different representations.^25^

### Characterizing Student Proficiency across Multiple Representations

Research in chemistry, physics, biology, and mathematics education has extensively documented students’ difficulties with representational competence (i.e., how to reason with representations) and, to a lesser extent, how to support student representational competence through various tools and interventions.^11,15,26–31^ Far fewer studies have characterized how students develop and use metarepresentational competence skills.^23,32,33^ Indeed, the Discipline-Based Education Research (DBER) report notes that representations are pervasive across STEM disciplines but highlights that major learning goals such as metacognitive coordination and transfer remain underexplored.^34^

Within organic chemistry, several studies over the past 15 years have characterized student reasoning with different organic chemistry representations. While reasoning with some representations (such as Lewis structures) has been well-documented in the literature,^7,15,35^ reasoning with others (such as chair conformations) remains relatively uncharacterized. With respect to Lewis structures, we know that fewer than half of organic chemistry students can spontaneously determine chemical and physical properties from them.^35^ We also know that students primarily use explicit and surface features when drawing chemical inferences from Lewis structures.^7^

A few studies have characterized student reasoning across multiple representations. One such study looked at students translating between wedge–dash diagrams, Newman projections, and Fischer projections. It concluded that feedback using molecular models provides better support for the development of student representational competence than exclusively verbal feedback.^36^ Another study examined how students interpreted wedge–dash diagrams and Newman projections, translated between wedge–dash diagrams and Newman projections, and generated and used Newman projections.^37^ This study suggests that students’ reasoning across these representations often relies on an incomplete interpretation of surface features and on algorithm-based translation strategies. Similarly, large-scale data collected using the Organic Chemistry Representational Competence Assessment (ORCA) across five institutions revealed strong interconnections among representational competence skills and that such skills cannot be assumed. These findings suggest persistent challenges in students’ representational competence across organic chemistry representations.

Further, individual studies on student reasoning with representations have largely focused on individual representations or small subsets of representations.^7,36,37^ To our knowledge, this is the first study within the DBER literature to comprehensively characterize student reasoning for such a large number of representations. Specifically, we examined how students perceive six commonly used molecular representations in organic chemistry, with each participant discussing a subset of representations and the six representations examined collectively across the full sample. Moreover, while prior studies have generated important insights into how students interpret, translate, and use individual molecular representations,^6,7,15,37–39^ little is known about how students identify the affordances and limitations of representations. Furthermore, relatively few studies have examined the role of heuristics in students’ reasoning about representations. Building on Talanquer’s call for a more explicit and granular characterization of reasoning with representations,^8^ this study investigates the reasoning patterns students employ to justify claims about their affordances and limitations.

### Research Questions

Building on prior research that identified gaps in our understanding of student reasoning with representations, we formulated two research questions:

- **RQ1**: What affordances and limitations do students identify across different molecular representations in organic chemistry?
- **RQ2**: What reasoning and argumentation patterns do students employ when identifying the affordances and limitations of different molecular representations in organic chemistry?

The first research question examines how students identify the affordances and limitations of common molecular representations in organic chemistry. A student’s understanding of affordances and limitations can either demonstrate representational reasoning (allowing for the interpretation of potentially relevant properties) or metarepresentational reasoning (highlighting an epistemic understanding of the purpose of each representation). In our analysis, we coded and tabulated the various representational affordances and limitations articulated by our participants. Findings addressing the first research question form the basis for Theme 1 (see the Results and Discussion and Conclusions sections).

The second research question explores the reasoning and argumentation patterns that students use to justify the affordances and limitations that they recognize. This analysis employed the analytical frameworks described in the following sections. Findings addressing the second research question form the basis for themes 2 and 3.

## Participants and Data Collection

### Setting and Participants

This study was conducted at a large public research university in the Southeastern United States. The participants recruited for this study were students in Organic Chemistry I, who had been taught and assessed (e.g., course homework, quizzes, and assignments) on the six representations by the time of data collection. Participants were recruited from four sections of Organic Chemistry I during the fall of 2020 and the spring of 2021. Each section was taught by a different instructor, all of whom used the same curriculum and textbook.^40^ Our sample size of twenty eight students allowed us to reach data saturation.^41^ Demographic information has been included in our Supporting Information (Table S11).

### Ethical Considerations

Approval from the Institutional Review Board was obtained before the start of the study to protect the rights and privacy of participants (IRB 20-0511 at UNC-Greensboro). Recruitment emails were sent to students detailing the voluntary nature of participation. Written informed consent was obtained from all of the participants prior to data collection. Participants also received detailed information about the study goals, the data collection process, and their right to withdraw from the study without penalty. Participants were assigned pseudonyms to protect their anonymity and received a $20 gift card each as compensation for their time.

### Data Collection and Interview Prompts

We used a semistructured, think-aloud interview protocol to collect data.^42^ The interview protocol described in this publication was part of a larger study designed, further described in our Supporting Information. Findings from other components of this project have been reported elsewhere.^7,18,37,39^

The protocol included a list of predetermined questions to guide the conversation with a flexible order of questions determined by the nature and content of student responses.^42^ The semistructured, think-aloud approach allowed us to ask follow-up questions and encourage participants to verbalize their thought processes to elicit their reasoning.^43^ Data were collected on Zoom, with each task (Table 1) presented on an individual PowerPoint slide. Participants used the Zoom annotation tool to highlight specific features of the representations being referenced. Audio recordings, video recordings, and screenshots of the participant drawings and annotations were captured. Data were transcribed verbatim, and student drawings were added to the cleaned transcripts.

**Table 1:**
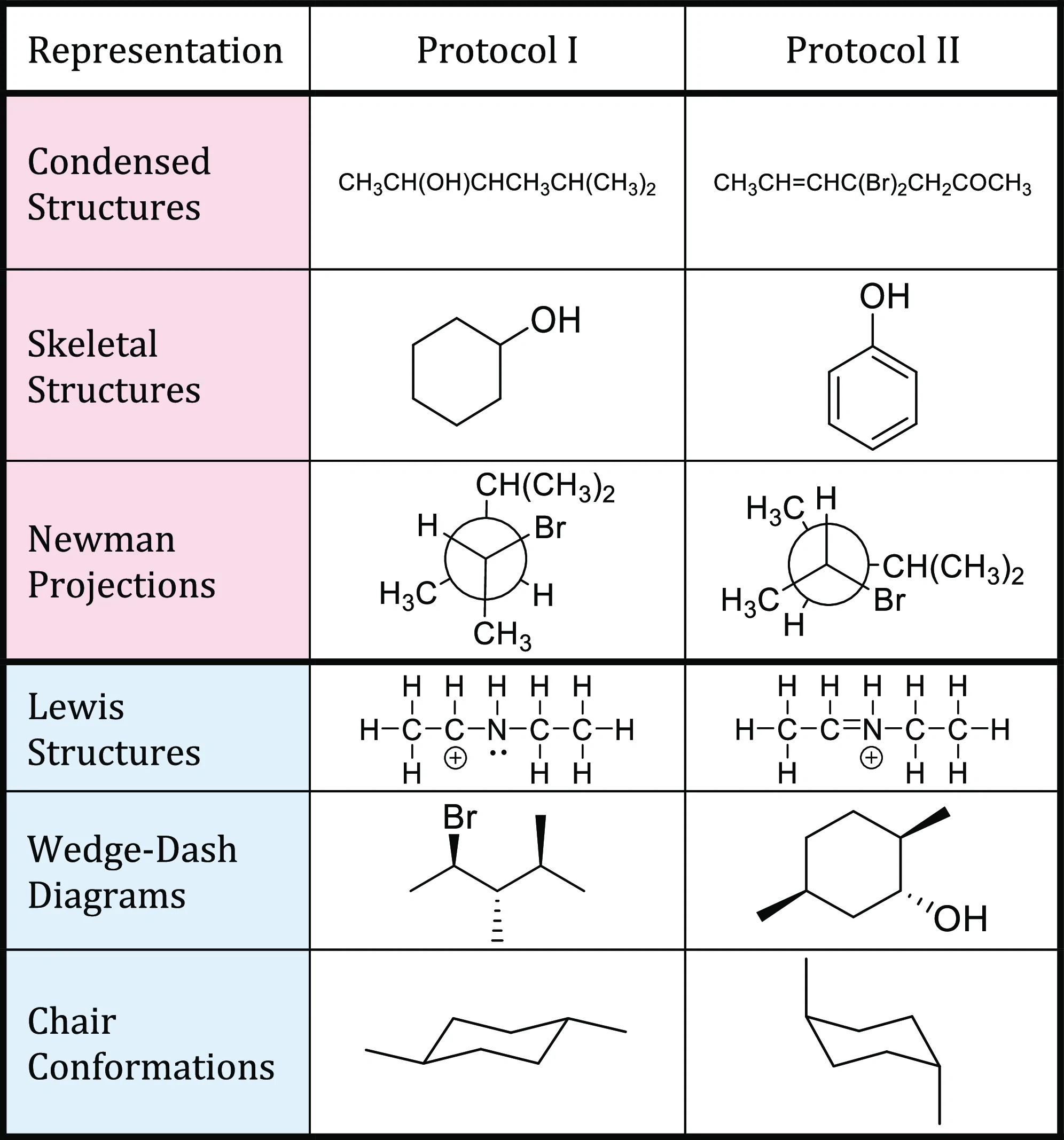
Structures Presented to Participants (*N* = 28) as Part of Our Qualitative, Semi-Structured, Think-Aloud Interview Protocol^a^

a The representations shaded in red were provided to Group A (*n* = 14) and those shaded in blue were provided to Group B (*n* = 15).

Twenty eight students participated in interviews involving commonly used molecular representations from the organic chemistry curriculum. Two molecules were selected for each representation, with each participant receiving one of the two possible molecules for each representation (either protocol I or protocol II, Table 1). This choice helped diversify representational features to elicit broader reasoning strategies while allowing us to examine whether patterns were robust across different molecular examples of the same representation.

The first participant was presented with all six representations from protocol I; however, this resulted in an excessively long interview. To reduce the interview length, the remaining participants were divided into two groups. Group A was presented with the condensed structure, skeletal structure, and Newman projection from either protocol I or protocol II. Group B was presented with the Lewis structure, wedge–dash diagram, and chair conformation from either protocol I or protocol II. The participant who received all six representations was included in both Group A (*n* = 14) and Group B (*n* = 15).

To circumvent potential complications from defining and explaining the terms “affordances” and “limitations” to participants, our protocol included the following questions and follow-up questions for each provided representation:

- What is the purpose of this representation?
- What does it tell you about this molecule (affordance)?
- What physical and chemical properties can you infer from this representation (affordances)?
- What does it not tell you (limitations)?
- What physical and chemical properties can you not infer from this representation (limitations)?
- Which representations do you prefer to use, and why?

## Analytical Frameworks and Data Analysis

### Data Analysis

Table 2 summarizes the codebook that we developed from various analytical frameworks and used to analyze our data. These frameworks are described in the subsequent sections.

**Table 2:**
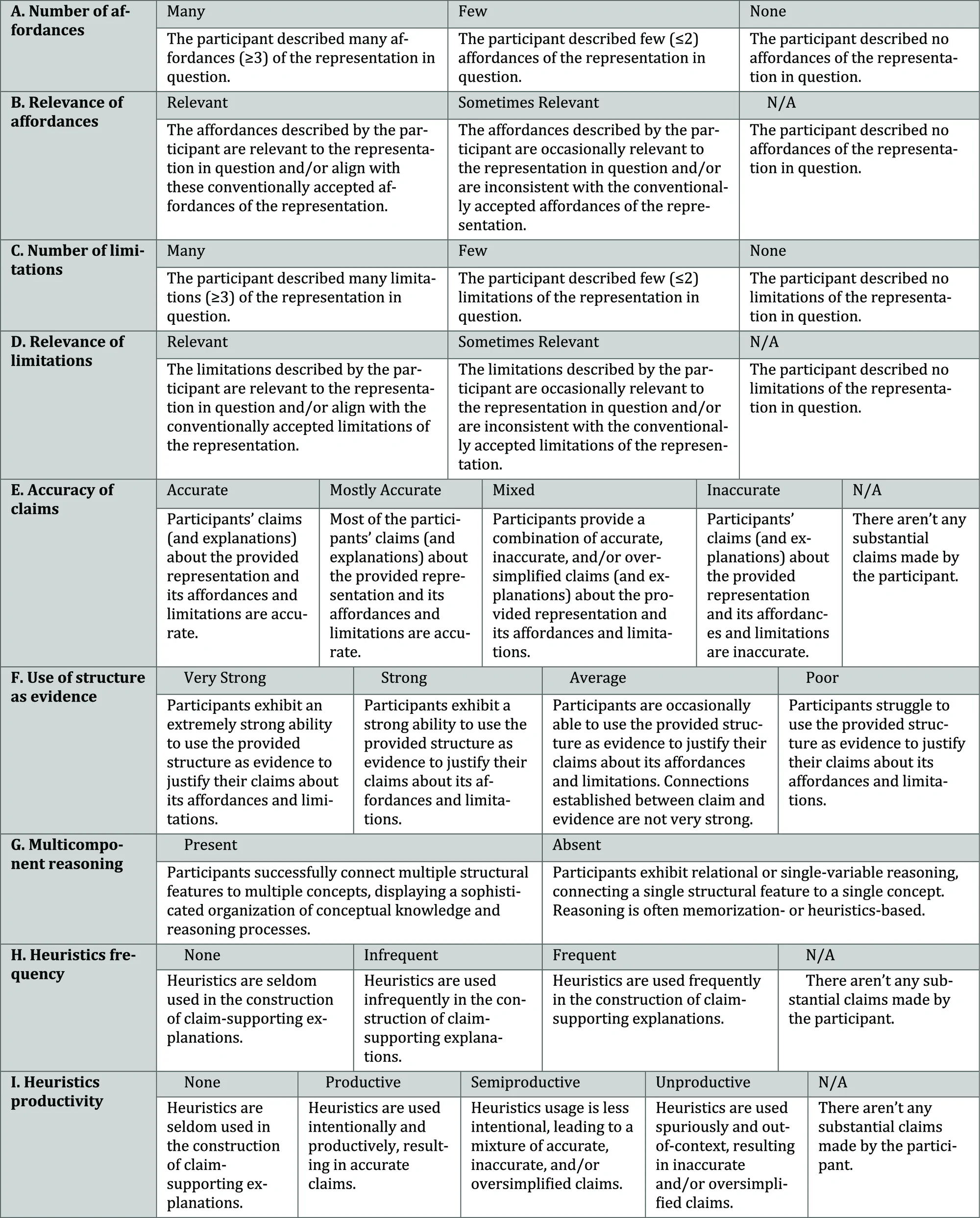
Codebook, Derived From a Combination of Various Analytical Frameworks, Used to Analyze Student Explanations of the Affordances and Limitations of Different Representations of Molecular Structure^a^

a Categories and codes are shown in gray, whereas code definitions are shown in white. See Tables S4, S5, and S7–S9 for representative example quotes.

To answer RQ1, concepts identified by participants as either an affordance or a limitation of a representation were inductively coded and grouped into categories. The number of affordances and limitations identified for each representation was tabulated across participants (rows **A** and **C** in Table 2). Most participants articulated 0 through 5 affordances and/or limitations, so ≥ 3 was chosen as our cutoff between few and many. Identified affordances and limitations were also grouped into relevant and nonrelevant, based on the conventionally accepted uses of each representation (rows **B** and **D**). Discussions between authors and with subject matter experts informed our coding of the relevance of affordances and limitations. For example, spatial orientation, bond angles, and bond rotation were considered relevant affordances of Newman projections, while formal charge and boiling point were not. Table S7 exemplifies the use of these codes in context.

To answer RQ2, participant explanations justifying an affordance or a limitation were deductively coded for claim accuracy (row **E**), the ability to use relevant structural features as evidence to support their claims (row **F**), the existence (or absence) of multicomponent reasoning (row **G**), the frequency of heuristic use (row **H**), and the productivity of heuristic use (row **I**). Coding of the data corpus was performed by using Microsoft Excel and Atlas.ti (v23).

To ensure the trustworthiness of our findings, RGB, AC, and SA collaboratively coded all the data and discussed every case of coding disagreement until consensus was reached.^44^ To synthesize patterns and central ideas in the data, RGB, AC, and MP held weekly meetings to engage in constant comparative analysis and thematic analysis.^45–47^ Throughout the analytic process, the authors maintained reflective memos to capture their thoughts about the data. The memos helped with communication between the authors about the coding process, facilitated the development of codebooks, and provided a way to document emergent patterns and themes in the data. Our record of memos bolsters the confirmability and dependability of the analysis,^48,49^ and frequent debriefing sessions between the authors furthered the credibility of our results by mitigating potential assumptions brought to the interpretive analysis.^46,50^

### Analytic Framework 1: Scientific Argumentation Patterns

In constructing an argument, scientists establish the acceptability of their explanations by coordinating supportive or contradictory evidence with a particular explanatory or descriptive claim for an observed phenomenon.^51^ For this reason, efforts to evaluate students’ explanations of scientific phenomena often focus on the development, strength, and structure of their arguments. Toulmin’s framework of argumentation was the first widely used argumentation framework in evaluating students’ scientific explanations.^51^ It describes the construction of a scientific argument as the process of using evidence, warrants, and backings to establish the validity of a claim (center of Figure 3).

**Figure 3:**
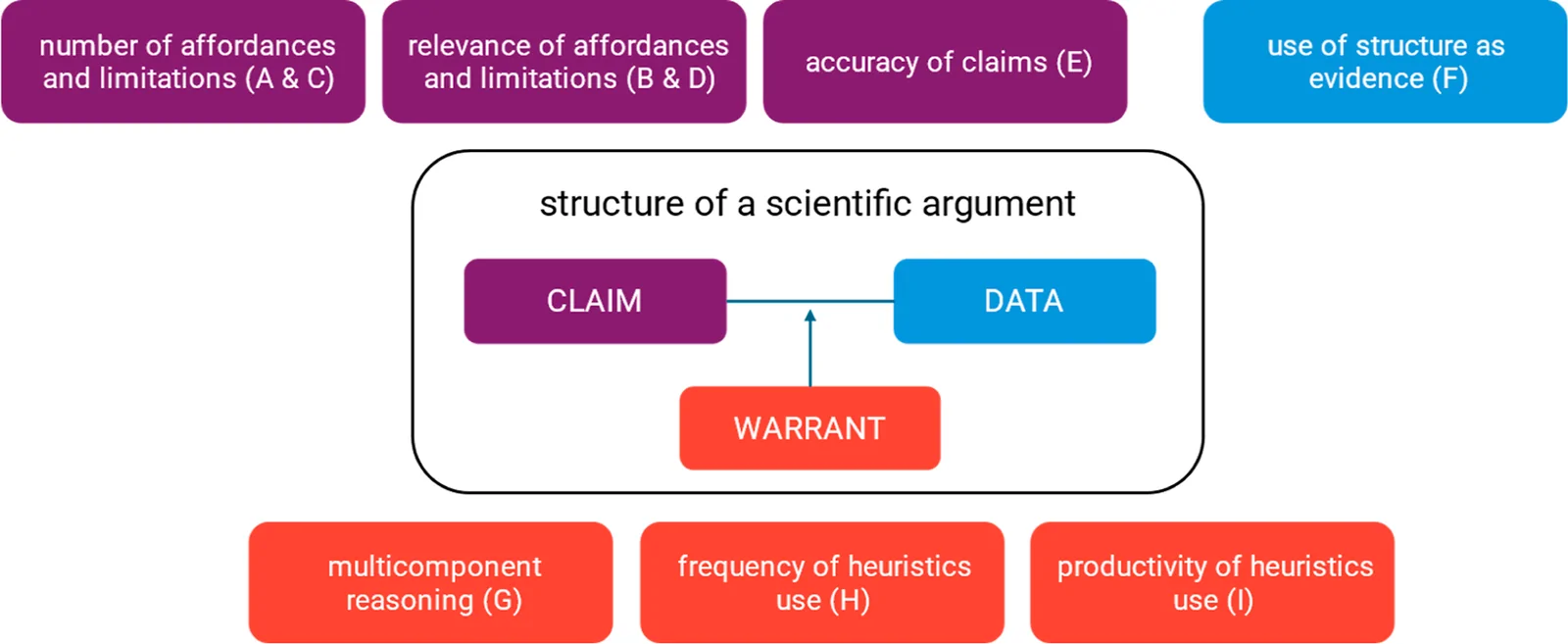
A summary of our analysis, structured around the three main components of scientific argumentation. Letters in the boxes correspond to the codebook in Table 2, where categories A–E were used to evaluate students’ claims, category F captured how students used structural data to support those claims, and categories G–I were used to characterize their representational reasoning (warrants).

While Toulmin’s framework evaluates an argument from a structural perspective (the field-invariant features), it fails to account for the appropriateness of claims, evidence, warrants, and backings in a discipline-specific context (the field-dependent features). To address these shortcomings, newer frameworks of scientific argumentation have been developed to ground the structure of argumentation in a field-dependent context and factor in the importance of multiple justifications, multiple sources of evidence, and scientific accuracy.^51–56^

In our analysis of students’ representational reasoning, we analyze students’ ability to support their claims with evidence drawn from the presented structures (Table 2, row **F**). This is field-independent and based on the number and strength of connections established between the claim and evidence. We also analyzed field-dependent aspects of student arguments, such as the contextual relevance of the affordances and limitations described (Table 2, rows **B** and **D**), claim accuracy (Table 2, row **E**), the existence (or absence) of multicomponent reasoning, and the presence (or absence) of heuristic-based reasoning (Table 2, row **H**). The subsequent sections further expand on multicomponent reasoning and heuristics. Figure 3 summarizes these analytical threads, organized around the three main components of a scientific argument.

### Analytic Framework 2: Multicomponent Reasoning

Chemical phenomena result from the complex interplay of multiple variables. Consequently, a comprehensive description and explanation of a chemical phenomenon requires understanding these different variables and their interactions. Multicomponent reasoning, as described by Sevian and Talanquer, is characterized by (a) the identification of salient entities within a system, (b) the recognition of explicit and implicit differentiating properties, (c) the identification of spatial and temporal connections between entities, (d) the invocation of relevant interactions between entities, and (e) the consideration and weighting of multiple variables.^57^ Several studies of student reasoning have demonstrated a lack of instructional support in developing multicomponent reasoning, with students often resorting to forms of single-variable reasoning, such as descriptive, relational, and linear causal reasoning.^57–60^

In our analysis of students’ representational reasoning, we often struggled to distinguish between descriptive, relational, and linear causal reasoning, primarily due to the brevity of some of the participants’ responses. Our primary aim was to identify participants demonstrating multicomponent reasoning, leading us to code student responses as either single-variable reasoning (encompassing descriptive, relational, and linear causal reasoning) or multicomponent reasoning (Table 2, row **G**).

### Analytic Framework 3: Dual Process Theory and Heuristics

Cognitive psychology research suggests that representational reasoning builds on two types of abilities: sense-making (or conceptual) competencies (Type 2 reasoning) and perceptual competencies (Type 1 reasoning).^61^ Sense-making competencies rely on field-dependent knowledge and skills (disciplinary conventions, known patterns, classification systems, procedural rules, chemical principles, and modeling primitives) that help connect visual features in a chemical representation to relevant concepts to build inferences about properties and behaviors.^8^ Perceptual competencies rely on the ability to quickly and effortlessly process meaning from visual cues in a representation and connect visual features across different types of representations.^8,62^ Perceptual competencies are automatic and fast and are strengthened through repeated exposure and experience working with representations. These shortcut reasoning strategies, as characterized by Talanquer among others, are called heuristics.^8,63,64^ They serve to minimize the cognitive load by either reducing the number of cues considered in making a judgment or providing rules of thumb for how and where to look for relevant information.^8,63^ Discipline-based education research across STEM fields has uncovered that novice learners often use heuristics (to varying degrees of productivity) when extracting information and making sense of visual representations. Experts also rely on heuristics, but disciplinary practice allows the deliberate, systematized, internalized, and productive activation of heuristics.^8,64–67^

To better understand how our participants perceived the affordances and limitations of multiple chemical representations, we characterized their underlying reasoning. Specifically, we identified and coded instances of heuristics-based (Type 1) reasoning in our participants’ explanations of the affordances and limitations of various chemical representations (Table 2, rows **H** and **I**). We identified the various types of heuristics used, tabulated the frequency of heuristic use for each participant, and coded instances of heuristic use as productive, semiproductive, and unproductive.

## Results

### Theme 1: Students Exhibit Baseline Proficiency in Identifying the Affordances and, to a Lesser Extent, Limitations of Multiple Molecular Representations.

Through inductive coding, we captured 42 codes of student-identified affordances and limitations of the six molecular representations, which were grouped into six broader conceptual categories: (i) structural properties (e.g., connectivity, composition, bond order, etc.), (ii) spatial properties (e.g., spatial orientation, substituent orientation, stereochemistry, etc.), (iii) electronic properties (e.g., formal charge, valence, electronegativity, etc.), (iv) energetic properties (e.g., steric effects, stability, ring strain, etc.), (v) physical properties (e.g., melting and boiling points, acidity or basicity, etc.), and (vi) practical considerations (e.g., simplicity, visualization effectiveness, etc.). Table 3 lists all 42 student-described affordances and limitations as well as their grouping into the conceptual categories. Also included in Table 3 are representative quotes that illustrate a student-identified affordance or limitation from each conceptual category. See Table S1 for the complete codebook that includes all code definitions and more example quotes.

**Table 3:**
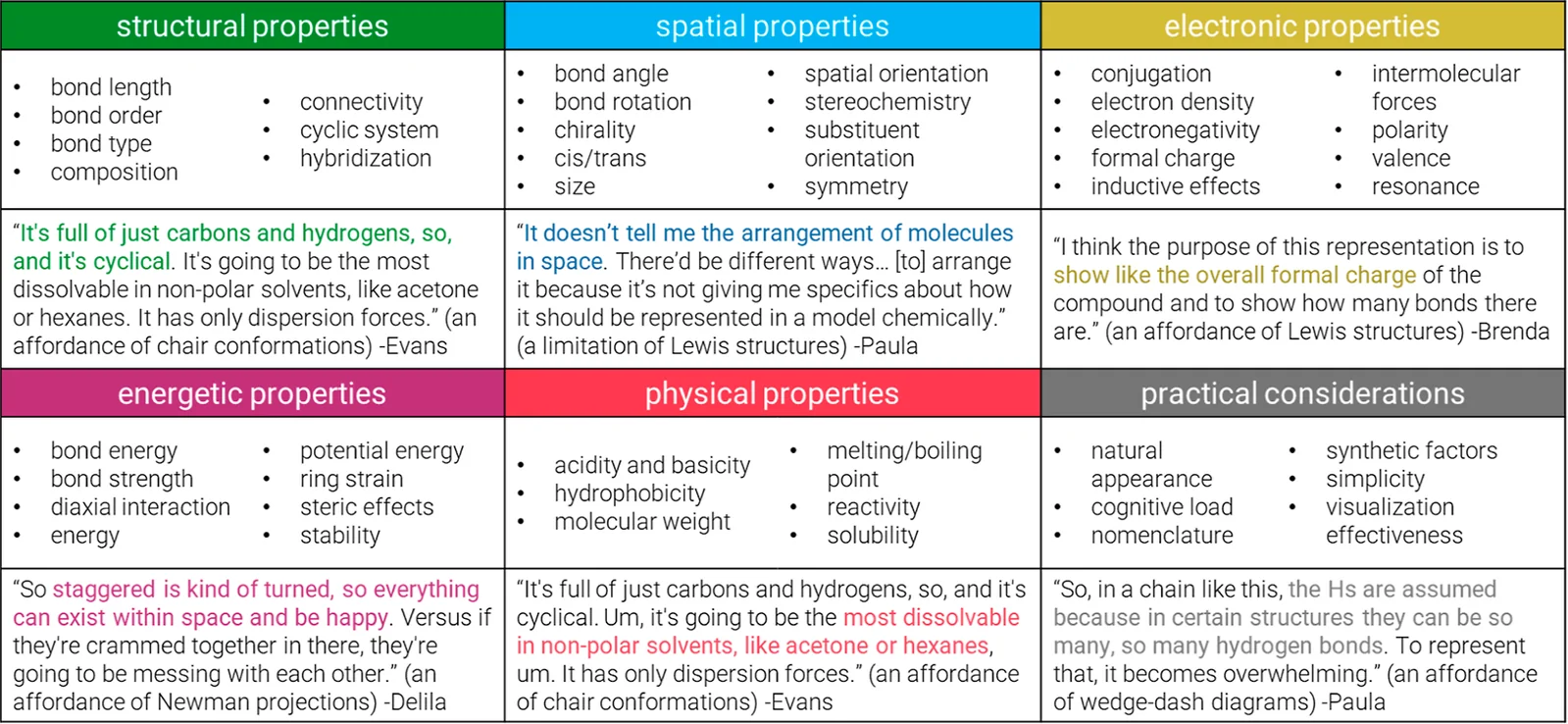
This Table Lists All Inductively Coded Affordances and Limitations Identified by Participants across All Six Molecular Representations^a^

a Affordances and limitations were categorized into six conceptual categories with a representative quote to illustrate a student-identified affordance or limitation from each conceptual category.

Many identified affordances and limitations reflected students’ ability to infer chemical properties from a representation (e.g., chirality, electronegativity, stability), whereas some reflected explicit evaluations of the representation itself (e.g., visibility of stereochemistry, simplicity, visualization effectiveness). The examples in Table 3 reflect different ways that students engaged with representational affordances and limitations. For example, in our representative example for structural properties, Evans used the chair conformation to infer physical properties of the represented molecule, including intermolecular forces and solubility. In doing so, he demonstrated representational competence by reasoning with the chair conformation to draw chemically meaningful conclusions. Through his inference, Evans demonstrates an implicit knowledge of some properties that can be derived from the representational features of a chair conformation (i.e., implicit affordances). On the other hand, in our representative example for spatial properties, Paula focused on information that a Lewis structure cannot communicate (i.e., a limitation). Rather than making inferences about the molecule itself, she evaluated the representation’s inability to convey three-dimensional spatial arrangement. This reflection about what a Lewis structure obscures demonstrates knowledge of the epistemic purposes and constraints of the representation (i.e., explicit metarepresentational reasoning). Despite attempted probing, a significant number of participants articulated chemical inferences that signified an implicit knowledge of the affordances and limitations of different representations (as exemplified by Evans), rather than an explicit awareness of the epistemic trade-offs and purposes of different representations (as exemplified by Paula).

The identified affordances and limitations within the conceptual categories are tabulated and plotted in Figure 4. See Table S2 for a more detailed summary of the most common codes across all six conceptual categories. As Figure 4 shows, participants articulated more affordances than limitations across all representations, suggesting that students are generally more proficient in articulating what they can infer from molecular representations than in recognizing what those representations do not convey.

**Figure 4:**
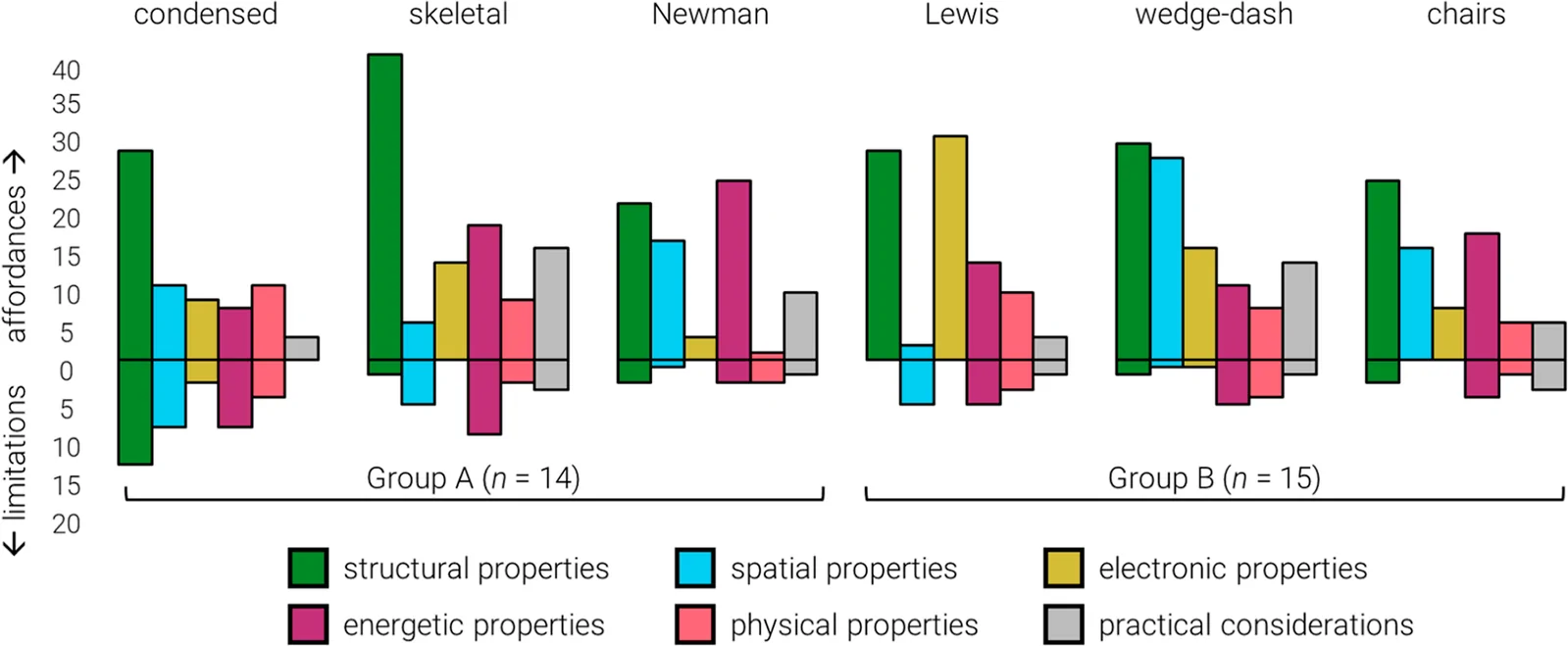
Conceptual categories of affordances and limitations identified by participants across the six representations. The positive *y*-axis represents the frequency of affordances described within a conceptual category, while the negative *y*-axis represents the frequency of limitations. Codes within each category are described and exemplified in Table S1. Participants in Group A (*n* = 14) were presented with a condensed structure, a skeletal structure, and a Newman projection, while those in Group B (*n* = 15) were presented with a Lewis structure, a wedge–dash diagram, and a chair conformation.

The more in-depth look at the six conceptual categories shows that students consistently identified structural properties, such as atomic composition and bond order, as key affordances across all representations. This is expected as each representation conveys at least some degree of structural information. Interestingly, structural limitations, such as reduced visibility of atomic connectivity, were mentioned only in relation to condensed structures, aligning with the commonly accepted view of this representation. Participants also associated specific chemical concepts with particular representations in ways that closely reflect expert consensus. For example, Lewis structures were noted for highlighting electronic properties like polarity, lone pairs, and formal charges. Wedge–dash diagrams were noted for showing spatial properties, especially stereochemistry and substituent orientation. Newman projections and chair conformations were identified as especially helpful for understanding steric interactions and relative stability, combining spatial and energetic insights. Finally, the practical considerations category included students’ reasons to use or not use a specific representation based on evaluations of what a representation reveals, obscures, or is useful for. For example, relative to other representations, some students criticized chair conformations for their visual complexity or skeletal structures for not showing all atoms explicitly. As such, many of these responses reflected elements of metarepresentational reasoning as students considered the relative strengths, limitations, and usefulness of different representations.

These findings suggest that students possess a foundational understanding of what common molecular representations communicate, particularly their affordances for reasoning about chemical structure and properties. It is encouraging to see that students’ descriptions show a notable degree of alignment with the perspectives of subject matter experts. This suggests that many students are beginning to develop an expert-like understanding of what different representations can reveal about chemical structures and processes. However, comparatively few students explicitly articulated representational limitations or reflected on the epistemic trade-offs involved in selecting representations. While some participants demonstrated explicit metarepresentational reasoning, most identified implicit chemical inferences, indicating that awareness of why particular representations are useful (or not) may remain an emerging aspect of representational and metarepresentational reasoning competence.

### Theme 2: Students’ Evaluation of Multiple Molecular Representations Is Often Supported by Memorization and Heuristics-Based Reasoning

#### Theme 2.1: Students Frequently Rely on a Range of Heuristics to Support Their Claims, with the Types and Frequencies of Heuristics Used Varying across Different Representations.

Unlike Theme 1, which examined participants’ perceptions of representational affordances and limitations, which involved both representational and metarepresentational reasoning, Theme 2 focuses primarily on how students used representations to make chemical inferences (representational reasoning). In our coding, we noticed a heavy reliance on heuristics^64^ that prompted us to delve into the reasoning patterns that our participants used when identifying representational affordances and limitations.

When tabulated across all six representations, we observed the repeated use of six of the ten reasoning heuristics,^64^ as well as of anthropomorphism^67^ (Figure 5). The most commonly used heuristic—generalization—occurs when individuals extend knowledge derived from a few familiar cases or rules to a new situation without carefully analyzing whether the same principles apply in that context. For example, when Ivy was analyzing the affordances and limitations of condensed structures, she stated the following about the physical properties of 4-methyl-2-pentanol: “Probably not going to be water-soluble because it’s got a lot of hydrocarbons, I guess. That’s all I can come up with from that.” Ivy noticed the hydrocarbon portion of 4-methyl-2-pentanol and immediately inferred that the molecule is not going to be soluble in water, even though it contains a polar hydroxyl (–OH) group and some branching, both of which promote partial solubility in water. Based on Ivy’s logic, all organic chemistry molecules should not be water-soluble because they contain carbons and hydrogens. In Ivy’s case, the generalization heuristic reflects an intermediate stage of understanding where she possesses fragmentary rules but has not yet fully developed multifeature representational reasoning. Definitions of other heuristics and example quotes illustrating their use can be found in Table S3. When looking for trends across the representations, we noticed that the nature, productivity, and context of heuristic use varied across the different representations provided (Figure 6 and Table S3). Heuristics were most frequently used in the context of skeletal structures and least frequently in the context of Newman projections. In addition to frequency, the types of heuristics used also varied across all six representations. The paragraphs below detail heuristic use for each representation.

**Figure 5:**
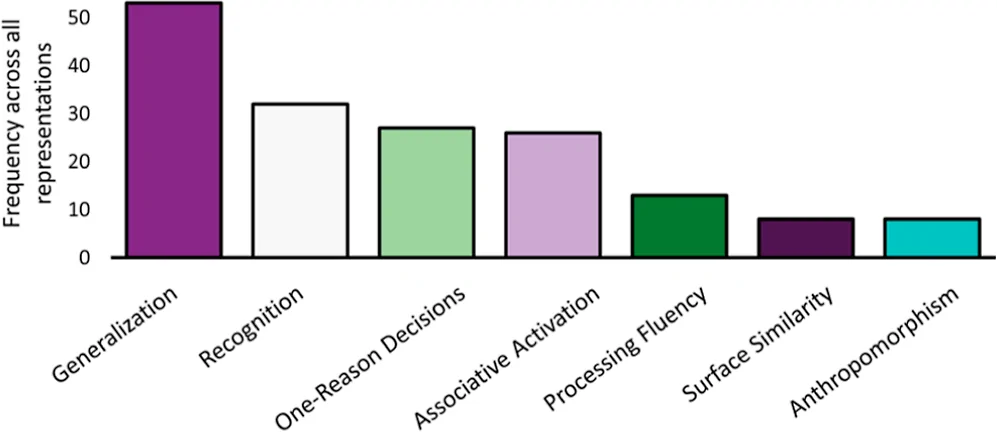
Cumulative frequency of heuristics used by participants across all six representations.

**Figure 6:**
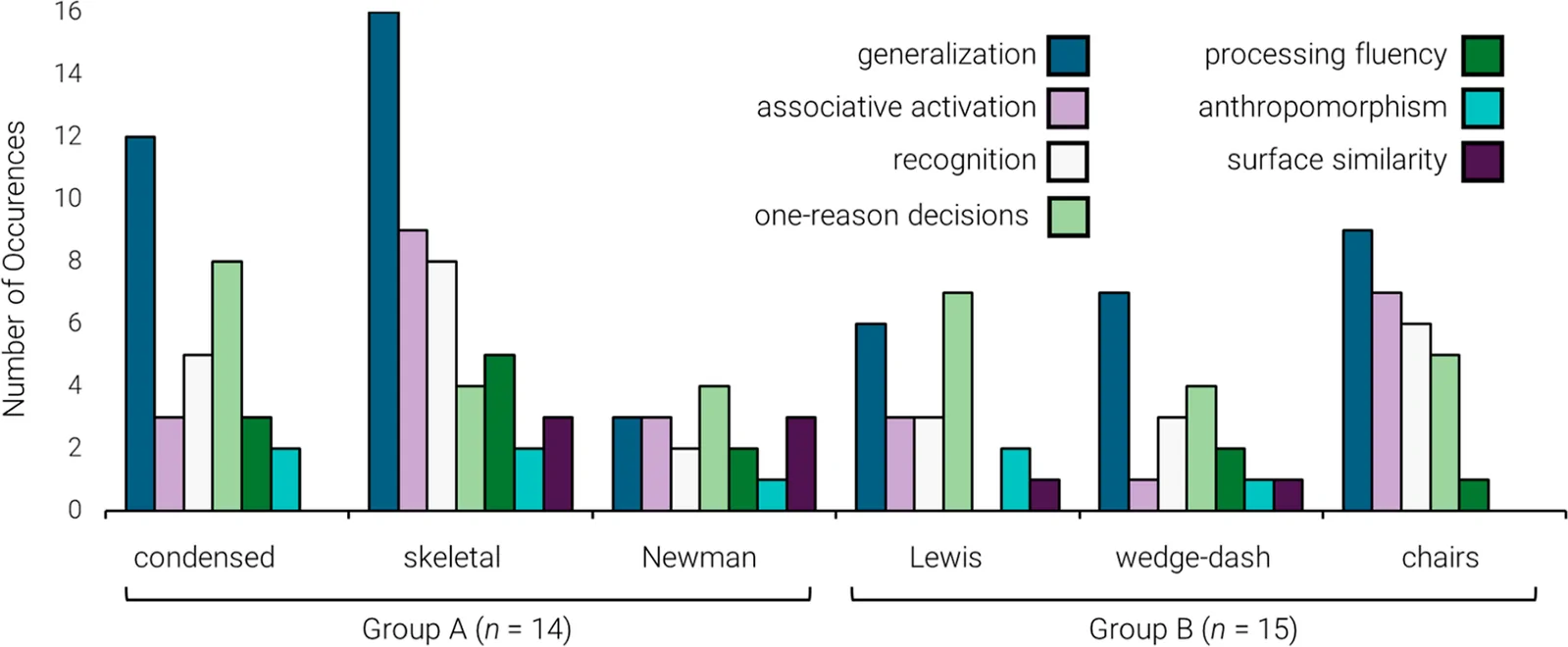
Frequency of the different types of heuristics used by participants across all six representations. Participants in Group A (*n* = 14) were presented with a condensed structure, a skeletal structure, and a Newman projection, while those in Group B (*n* = 15) were presented with a Lewis structure, a wedge–dash diagram, and a chair conformation.

##### Condensed Structures

When provided with the condensed structure of the alkene, several participants used the generalization heuristic to explain the influence of the double bond on the molecule’s stability and reactivity. Participants who received the condensed structure of the alcohol used generalizations to explain the influence of the hydroxyl group on polarity, chemical interactions, and boiling point. Participants used the recognition and associative activation heuristics to make inferences about the chemical properties of alcohols from their real-life experiences with various alcohols. Such inferences were often vague and oversimplified, which could be attributed to the inherent lack of structural features in condensed structures. For example, Isabel commented on solubility, saying that “the best way for me to describe [solubility] is when I think of alcohol and water, they don’t mix at all. The best way I remember it is by remembering real-life instances.” Her reasoning reflects associative activation, where prior real-life experiences are automatically retrieved and used to make chemical inferences, without careful analysis of the chemical structure. In doing so, she also demonstrates the generalization heuristic, extending a broad pattern to a context where it does not universally apply. The lack of structural details in condensed structures also resulted in assumptions about atomic connectivity and the impact of connectivity on molecular stability. Some participants used the apparent physical length of the condensed structure on the screen as a predictor for properties such as melting and boiling points.

##### Lewis Structures

Participants relied on fewer heuristics when making inferences from Lewis structures, which can be attributed to the higher amount of structural information that Lewis structures encode, relative to condensed structures. On seeing either the double bond or the positive charge (depending on the structure presented), several participants used heuristics (generalizations, associative activations, and one-reason decision making) to draw noncausal inferences about the molecule’s reactivity, valence, and bonding properties. A few participants used anthropomorphisms to describe what makes a carbon “happy” (i.e., the conditions that satisfy a carbon’s octet) from an electronic perspective. Some participants used one-reason decision making, reporting symmetry, intermolecular forces, or the degree of structural branching as the only factor that influences physical properties.

##### Skeletal Structures

The aromatic system present in one of the structures triggered both the productive and unproductive use of associative activation and recognition. Many participants instantly recognized the benzene ring and made quick inferences about bond order, conjugation, stability, and hybridization (with varying degrees of accuracy), often relying on their memory of aromatic systems instead of the presented structure. A few participants conflated cyclohexyl and phenyl systems, exemplifying the surface similarity heuristic. Participants made both accurate and inaccurate generalizations about the properties of cyclic systems (involving conformational dynamics, energetics, and stability), often without relying on structural evidence. Like with condensed structures, some participants noticed the hydroxyl group and relied on associative activation, recognition, and generalizations to arrive at often vague or incorrect inferences about properties of the given molecule, such as acidity, solubility, and reactivity. Meghan commented on the fact that “[the represented molecule] probably has a high boiling point cause it’s big.” This quote clearly reflects a one-reason decision making heuristic, where an individual bases a decision or conclusion on a single salient cue (in this case, molecular size) while ignoring other relevant structural or chemical variables that jointly determine the property in question.

##### Wedge–Dash Diagrams

Many participants relied on recognition and memorization to make inferences about the three-dimensionality of molecules, having internalized (in some cases incorrectly) associations among wedge/dash, cis/trans, and in/out. Participants often relied on generalization, recognition, and one-reason decision-making to make inferences about the effect of substituents and symmetry on the stability and energetics of molecules. As with other representations, participants noticed the hydroxyl or bromine group, which triggered associative activation, generalizations, recognition, and one-reason decision making, leading to vague or noncausal inferences about physical and chemical properties like polarity, acidity, melting and boiling points, and reactivity. A few participants struggled with processing fluency and could not extrapolate implicit hydrogens, with some believing that unlabeled methyl groups were implied hydrogens.

##### Newman Projections

Participants used fewer heuristics with Newman projections relative to other representations, often making successful interpretations about their relative energy and stability. Participants broadly supported their claims about energetics and stability with structural evidence, but a few relied solely on their memorized recognition of “good” and “bad” steric interactions. Some participants relied on one-reason decision making, factoring in either steric bulk or absence of double bonds as the only variable in their conformational and energetic analyses. For example, in the words of Nadia, “I’m not seeing any double bonds, [and] seeing one bromine group. So, [this molecule is] not going to have high energy.” Some participants used anthropomorphisms to support their understanding of steric interactions, as in the case of Delila, who commented on conditions for a molecule’s happiness and compared steric repulsion to siblings packed into the backseat: “So staggered is kind of turned, so everything can exist within space and be happy. Versus if they’re crammed together in there, they’re going to be messing with each other, like siblings in the backseat.” In this quote, Delila uses an anthropomorphism to help her visualize an abstract concept, such as steric repulsion, through an analogy that is familiar to her. Additionally, a few participants struggled with processing fluency, having internalized highly algorithmic methods of interpreting Newman projections.

##### Chair Conformations

Through generalizations and one-reason decision-making, many participants provided noncausal and oversimplified explanations about the relative stability of chair conformations based on observed axial and/or equatorial substituents. Many participants demonstrated associative activation and recognition in their assumption that symmetric substituents around a cyclohexane ring (regardless of whether they are equatorial or axial) contributed to the increased stability of a substituted cyclohexane by “balancing [the ring] out.” Relative to the other two-dimensional representations, participants recognized that one could make inferences about relative stability using chair conformations. A few participants relied on their recollection from class that chair conformations assist with interpretations about relative stability, but these participants struggled to make or justify specific energetic claims about the molecules they were provided with.

Overall, heuristic use was most prevalent when representations lacked explicit connectivity, composition, or spatial information (e.g., condensed and skeletal structures) and diminished when representations showed three-dimensional features more explicitly (e.g., Newman and wedge–dash). These findings suggest that representational explicitness influences whether students engage in heuristic-based (Type 1) reasoning.

#### Theme 2.2: Student Use of Heuristics in Supporting Chemical Claims Can Be Either Productive Or Unproductive, with Productivity Emerging When Heuristics Are Supported by Causal and/or Multicomponent Reasoning.

In addition to identifying the types of heuristics used, we coded participant use of heuristics as productive, semiproductive, or unproductive, based on the accuracy of the associated claims. For an example of a productive heuristic, we can look at Karen’s interpretation of the skeletal structure in Protocol B, in which she sees a double bond and knows “that [carbon] is not a full sp^3^, it’s sp^2^.” Her identification of a double bond in the structure triggers her recognition of carbon being sp^2^ hybridized, making this an example of associative activation. Though this method of determining hybridization is algorithmic and heuristics-based, it is productive in the given context and is often used by experts as a quick way of predicting hybridization. Conversely, an example of an unproductive heuristic was Teresa stating that “[The Newman projection] is probably like... a pie graph, but I don’t know much else about it.” This is an example of surface similarity as Teresa sees the trisected circle within the Newman projection and thinks of a pie chart. This is an unproductive heuristic as her recognition of the superficial similarity between a Newman projection and a pie chart impedes any further interpretation of the Newman projection. When looking at a Newman projection, Nadia concludes that the structure is “not going to have high energy” because she “is not seeing any double bonds [and] is seeing one bromine group.” While bond order and atomic composition do impact a molecule’s potential energy, Nadia’s generalization about energetic factors is not particularly intentional, leading to an oversimplified albeit not inaccurate claim. This led us to categorize such instances of heuristic usage as semiproductive. See Table S4 for code definitions and examples regarding the productivity of heuristics.

The frequency of the heuristics used by students did not predict the productivity of their reasoning and the accuracy of their claims. For example, some students who used many heuristics (e.g., Nate, Will, Irsa) made mostly correct, scientifically sound claims, while others who used just as many heuristics (e.g., Maggy, Isabel) made primarily inaccurate claims. Similarly, some students who used very few heuristics (e.g., Evans, Charlie) still made accurate claims, while others with equally few (e.g., Sybil, Edith) made inaccurate ones. In other words, using more or fewer heuristics did not determine the quality or accuracy of the students’ claims. See Table S5 for code definitions and examples regarding the accuracy of claims and Table S6 for the frequency and overall productivity of heuristics used by each participant.

While analyzing our participants’ explanations, we also examined their ability to use multiple implicit and explicit structural features as causal or supporting evidence for their claims (Table 2, row F and Table S8 for example quotes), which ranged from very strong to poor, and noted the presence or absence of multicomponent reasoning (Table 2, row **G** and Table S9 for example quotes). We observed a high co-occurrence between the productive use of heuristics, the strong use of implicit and explicit structural features, and the presence of multicomponent reasoning. In other words, students who could draw on multiple types of structural evidence and reason across multiple interacting variables were more likely to use heuristics productively. Conversely, we also observed a high co-occurrence between the unproductive use of heuristics, a poor use of implicit and explicit structural features, and the presence of single-variable reasoning. These results suggest that the ability to identify structural features and the presence of multicomponent reasoning are better indicators of the productivity of heuristics than the frequency of heuristics use.

### Theme 3: Students with Stronger Reasoning, Characterized by Multicomponent Reasoning and/or Productive Use of Heuristics, Consistently Used Structural Evidence to Support More Accurate Claims and Identified a Higher Number of Relevant Affordances and Limitations.

Through constant comparative analysis of coded affordances and limitations as well as of participants’ underlying reasoning, we were able to identify patterns across participants and across representations. Through our analysis, we observed five distinct qualitative tiers of reasoning within each representation, with cross-representational parallels across each tier. Our analysis was supported by reflective memoing and discussions within the coding team. Importantly, tiers (and student placement into tiers) varied for each representation as students did not always reason with the same level of sophistication across all representation types. For example, Paula was placed in tier 2 for her reasoning with Lewis structures and wedge–dash diagrams but in tier 4 for chair conformations, suggesting that representational competence skills are context-dependent (see Table S10). To simplify and highlight major trends, we aggregated tier patterns across representations and visualized them, shown in Figure 7. This figure illustrates how students’ claims about representational affordances and limitations, their use of structural evidence, and the quality of their warrants collectively distinguish the five reasoning tiers.

**Figure 7:**
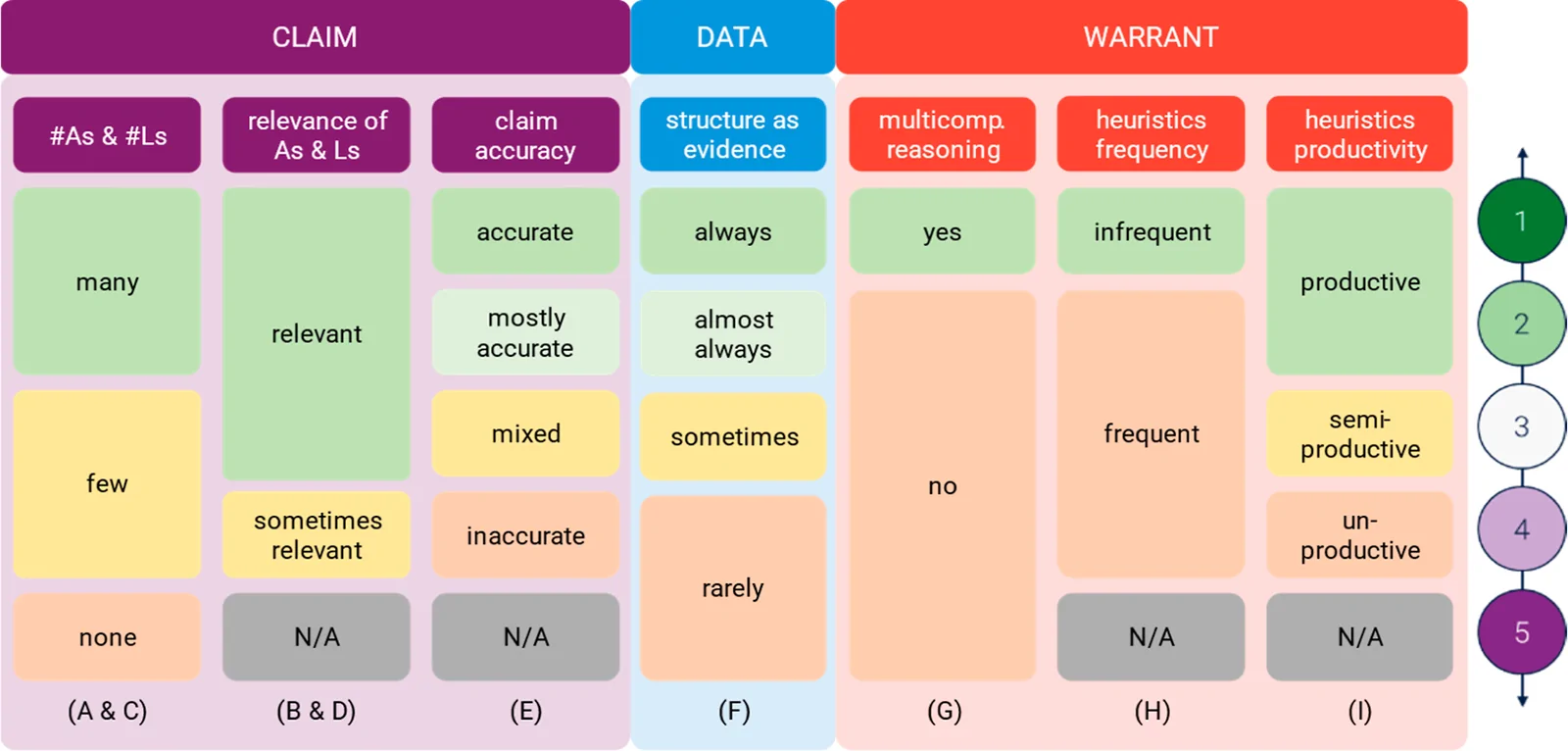
Cross-representation synthesis of the five tiers of students’ representational reasoning. The figure shows how codes from Table 2 (A–I) align across the five tiers and are organized around the claim, data, and warrant components of scientific argumentation. The size of each box represents how many tiers that box spans. As an example, in the column titled #As & #Ls, “many” spans tiers 1 and 2, “few” spans tiers 3 and 4, and “none” spans tier 5. Table S10 provides the more granular findings across each representation that informed this synthesis. Note that “As” = affordances and “Ls” = limitations. “N/A” indicates cases where coding was not possible because students’ responses were too brief or unclear, except for heuristic productivity in tier 1, which could not be coded since those students did not use heuristics.

Participants in tiers 1 and 2, representing the strongest reasoning with representations, either avoided heuristics altogether or used them infrequently but productively. Students in these higher tiers also showcased multicomponent reasoning because they used structure as evidence to support their claims. As a result, these students identified a higher number, more relevant, and more accurate affordances and limitations of various representations. In contrast, participants in tiers 4 and 5, who showed the weakest reasoning with representations, are characterized by either too brief or unclear responses that could not be coded or responses that showcased an absence of multicomponent reasoning and a frequent reliance on unproductive heuristics. These students rarely used structural features as evidence to support their mostly inaccurate claims and struggled to identify representational affordances and limitations. In addition to the number and relevance of articulated affordances and limitations, we observed trends in the number of conceptual categories (e.g., structural properties, spatial properties, electronic properties, energetic properties, physical properties, and practical considerations) across which participants articulated affordances. As depicted in Figure S1, participants in tier 1 articulated affordances across a wider range of categories, while participants in tier 5 focused on a narrower subset, often limited mainly to basic structural features. Taken together, these results build on Talanquer’s work, suggesting that strong representational reasoning is closely linked to making scientifically accurate claims and using structure-based inferences, and vice versa.^8^

## Limitations

This is a qualitative study with a sample size of 28 students from a single institution. While we do not make generalizability claims due to the qualitative nature of the study, the contextual detail provided here enables readers to evaluate the transferability of these findings to other organic chemistry or STEM learning contexts that involve representational reasoning.^68,69^ To address the concerns around generalizability, future research could triangulate detailed qualitative data with quantitative data from assessment instruments such as the Organic Chemistry Representational Competence Assessment (ORCA).^18^

While semistructured, think-aloud interviews provide deep insights into students’ reasoning, our analysis might be limited to what students choose to verbalize. We might have overlooked some cognitive processes and explicit features of representations that participants used but chose not to verbalize, even though they were encouraged to think aloud.

All interviews were conducted and recorded virtually (due to the pandemic), which limited our ability to fully capture student gestures. Gestures can play an important role in tasks that require spatial reasoning, which can be the case for representations that communicate 3D information (e.g., wedge–dash diagrams, Newman projections, and chair conformations).^70,71^ Without a complete record of these nonverbal behaviors, our analysis does not capture the role gestures play in supporting or externalizing students’ reasoning processes.

Another limitation concerns the use of different molecular examples across the six representations, which introduce a potential confounding variable. While this design choice was intentional (e.g., to diversify representational features to elicit broader reasoning strategies), it is possible that molecular differences influenced the student reasoning. However, even though each representation featured a different molecule, the molecules presented to participants across representations are common prototypical examples, often used to illustrate that specific representation in context (e.g., 1,4-dimethylcyclohexane is a prototypical example to illustrate chair conformations). Furthermore, findings were corroborated across two different molecules for each representation, allowing for richer insights and more robust patterns.

Finally, our interviews capture students’ ability to identify the affordances and limitations of representations at a single moment in time. We do not account for how this skill might develop or shift over the course of the organic chemistry course sequence.

## Discussion and Conclusions

This study contributes to a growing body of literature on representational competence and reasoning in organic chemistry by characterizing how students reason about the affordances and limitations of six common molecular representations. In doing so, it responds to Talanquer’s call for a more explicit and granular characterization of reasoning with representations.^8^

To characterize student reasoning and proficiency with the affordances and limitations of multiple representations, we conducted qualitative, semistructured interviews with 28 Organic Chemistry I students, probing both the identification of affordances and limitations across multiple representations, as well as the various reasoning and argumentation patterns employed by students across multiple representations. This work expands on the field’s growing awareness of the inextricable link between representational competence and reasoning.^6,8,27,72^ Further, this is one of the first characterizations of how students engage with the representational affordances and limitations of multiple molecular representations.

Our analysis revealed that students exhibit baseline proficiency in identifying the affordances and, to a lesser extent, limitations of multiple molecular representations in organic chemistry. In other words, students were stronger at articulating what a representation can convey than they were at articulating what a representation cannot convey. Students successfully identified structural properties across all representations, as well as electronic properties within Lewis structures, spatial properties within Newman projections, wedge–dash diagrams, and chair conformations, and energetic properties within Newman projections and chair conformations. Encouragingly, many students identified affordances and limitations that closely aligned with the perspectives of subject matter experts, suggesting that they had developed a meaningful understanding of what different molecular representations can and cannot communicate.

Furthermore, some students went beyond using representations to make chemical inferences and explicitly reflected on the strengths, limitations, and contextual usefulness of the different representations. These instances of metarepresentational reasoning provide additional evidence of developing expertise as some students demonstrated not only the ability to reason with representations but also an awareness of why particular representations may be more or less useful for specific purposes. While a few students demonstrated such metarepresentational reasoning, most students articulated chemical inferences from the provided molecular structure rather than explicitly evaluating the epistemic features of the representation that facilitate contextual chemical interpretation. Our findings are consistent with the previous literature suggesting that metarepresentational competence receives relatively little explicit attention in postsecondary chemistry education.^16,17,22,23,25^ If students are rarely asked to reflect on why particular representations are useful, what information they obscure, or when one representation should be preferred over another, it is perhaps unsurprising that relatively few participants spontaneously engaged in this form of reasoning.

This baseline proficiency with the affordances and limitations of multiple molecular representations is often supported by memorization and heuristic-based reasoning for most of the students.^8,64,67^ Specifically, students often rely on generalization, recognition, one-reason decision-making, and other associative or simplifying heuristics to support their claims about molecular structure. Further, heuristic use can be productive or unproductive, with productive heuristics often co-occurring with causal and/or multicomponent reasoning.^57^

The context in which students employ heuristics varies across the representations. Overall, heuristic use was most prevalent when representations lacked explicit connectivity, composition, or spatial information (e.g., condensed and skeletal structures) and diminished when representations showed three-dimensional features more explicitly (e.g., Newman and wedge–dash). These findings suggest that representational explicitness influences whether students engage in heuristic-based (type 1) reasoning. One possible reason is that condensed and skeletal structures impose a high intrinsic cognitive load,^8,58^ where students must infer connectivity, geometry, and/or implicit features. To manage this, they default to fast, associative reasoning (type 1) rather than more effortful, analytical reasoning (type 2).^61,64^ Another possible reason is that condensed, skeletal, and Lewis structures are introduced early and heavily reused, so students develop pattern recognition and shorthand associations. In contrast, Newman projections, which are less familiar and more complex, are often explicitly taught with step-by-step reasoning (e.g., steric analysis and torsional strain diagrams), encouraging more systematic (type 2) processing.

Finally, we identified five tiers in student reasoning about the affordances and limitations of organic chemistry representations. Students among the more proficient reasoning tiers have stronger reasoning abilities (as exemplified by multicomponent reasoning and/or the use of productive heuristics), make more accurate claims about the affordances and limitations of representations, identify affordances across more conceptual categories, and identify more relevant affordances and limitations.

However, students among the developing reasoning tiers make more inaccurate claims about the affordances and limitations of representations, identify affordances across fewer conceptual categories, and often identify irrelevant affordances and limitations. Overall, progression across the tiers involves shifting from single-component, heuristic-driven claims to multicomponent, evidence-based arguments that include explicit warrants linking representational features to chemical concepts and phenomena.

Studies characterizing representational reasoning across multiple (more than 2–3) representations are scarce, with his work contributing to our growing knowledge about representational reasoning. Further, this work adds to the growing body of literature that suggests that the constructs of representational competence, conceptual understanding, and chemical reasoning are closely linked and highly interdependent.^6–8,37^ Specifically, our results suggest that student proficiency with the relative affordances and limitations of multiple molecular representations is intricately intertwined with attributes of student reasoning, such as multicomponent reasoning and the productivity of heuristics.

## Implications

### Implications for Researchers

Prior studies have elucidated a lack of instructional and textbook support for representational competence.^16,17,24,25^ In corroboration, our study demonstrates a reliance on heuristics when students engage in representational skills, such as describing the relative affordances and limitations of multiple representations. A useful next step is to collect classroom observation and course artifact data to understand what instructional supports could help scaffold representational reasoning.

This study highlights the need to further characterize areas of student representational competence. Specifically, our results underscore the need to characterize the interplay between various representational competence skills, such as the ability to identify affordances and limitations, translate, interpret, and generate.^18^ Little is known about how proficiency (or lack thereof) in one of these skills affects proficiency with others.

In this study, we elucidate the relationship between student representational competence and attributes of student reasoning, such as the existence (or lack thereof) of multicomponent reasoning^57^ and the degree of productivity of the use of the heuristics.^64^ This characterization brings us closer to a unified model of learning in organic chemistry that centers the complex interplay between representational competence and reasoning at the forefront of student learning in organic chemistry.^6^ There are opportunities to elucidate further the relationship between student representational competence and student reasoning with other potentially relevant constructs, such as scientific modeling,^73–75^ visuospatial ability,^76^ and science and engineering practices.^77,78^ For example, this study highlights the need for additional research explicitly focused on students’ metarepresentational competence. While we observed instances in which students reflected on what representations reveal, obscure, or are useful for, our interview protocol was not designed to systematically characterize metarepresentational competence. Future work should elicit students’ reasoning about the epistemic purposes, trade-offs, and contextual appropriateness of different representations. Such work would help clarify how students learn not only how to reason with representations, but also why and when particular representations are most useful in supporting chemical thinking.

Additionally, in Figure 7, we use the term “tiers” to reflect the qualitative progression in performance and reasoning observed across our participants. However, these tiers should be interpreted as a synthesis of qualitative findings rather than a formally validated hierarchical model. Future work should examine whether this proposed progression is supported in larger samples using quantitative approaches.

### Implications for Practitioners

This study has revealed that students possess baseline proficiency in identifying the affordances and limitations of different representations used in a typical organic chemistry classroom. When probed about these affordances and limitations, the majority of students articulate various chemical inferences instead of evaluating the epistemic features of a representation that facilitate chemical inference. Further, a significant portion of this baseline proficiency can be attributed to memorization and heuristic-based reasoning.

Students know representational affordances but often struggle when making causal chemical inferences. This study identifies areas of representational competence that require more instructional support and scaffolding in the classroom. Through various methods of epistemic signaling, such as (a) the simultaneous (rather than sequential) introduction of multiple representations,^79^ (b) frequent use of case comparisons,^80^ and (c) the inclusion of problems in quizzes and tests that explicitly address the affordances and limitations of multiple representations (such as problems that probe how students justify their claims and what evidence they draw from representations), instructors can support student representational competence. Importantly, this does not require us to add more content or spend extra time on representations—representations are already central to our courses. The key is to make more strategic, evidence-based choices about how to use class time.

Assessment should reinforce these goals. For example, assessment tasks written in the claim–evidence–explanation format, where evidence comes from structural features of representations, can encourage students to practice argumentation.^51^ Furthermore, assessment items can be designed to foster critical engagement with the affordances and limitations of multiple representations, based on a provided chemical context. Based on our results, we designed sample assessment items that center the aforementioned design considerations (Figures 8 and 9). These questions explicitly probe students’ ability to compare the affordances and limitations of different representations within a relevant context. The questions’ epistemological messaging encourages the interpretation of both explicit and implicit structural features, causal multicomponent reasoning, and the construction of sound scientific arguments. Feedback should go beyond right or wrong answers to highlight reasoning strategies and help students refine their approaches.^7,37^

**Figure 8:**
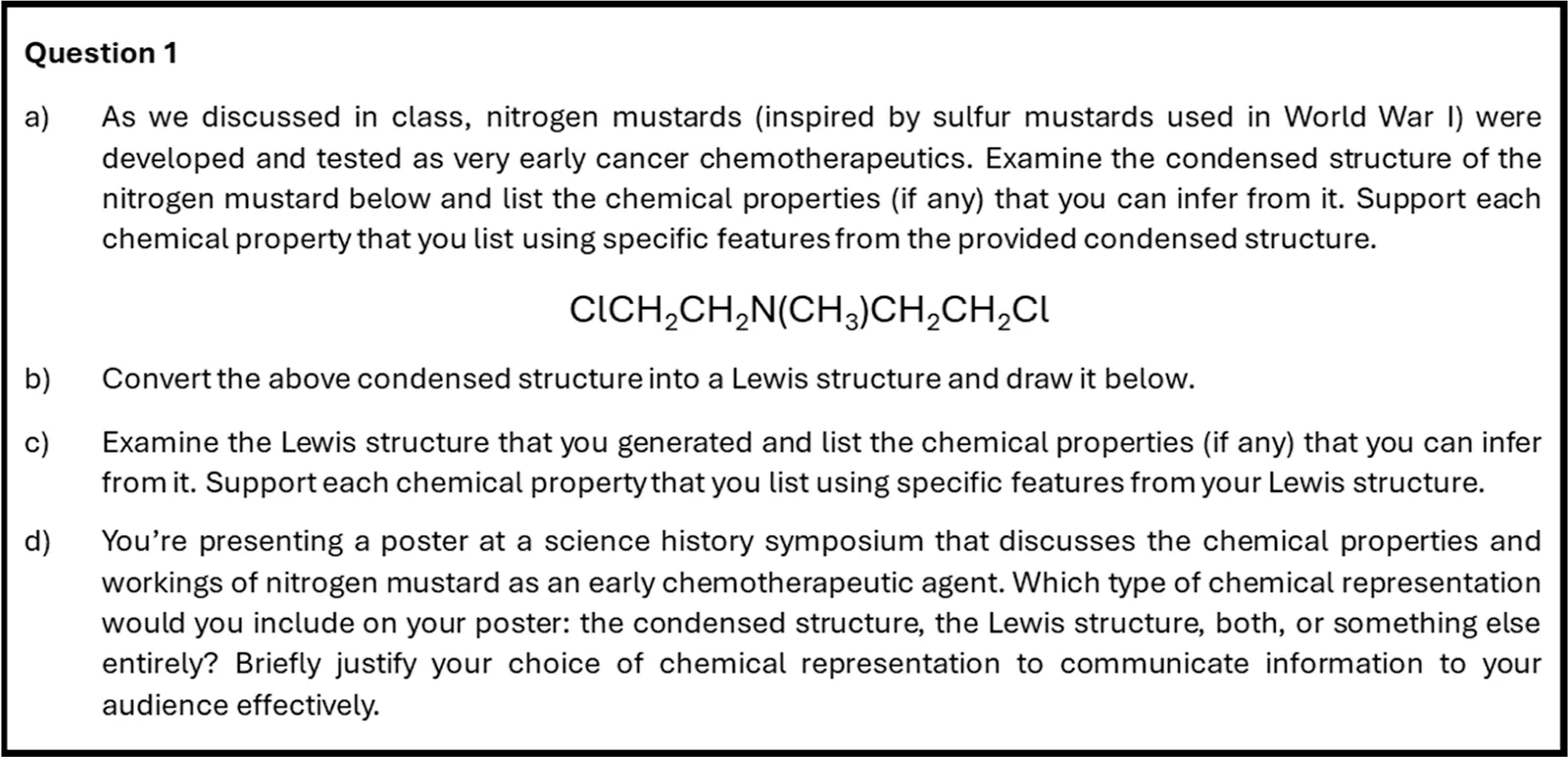
A scaffolded assessment item that explicitly encourages reasoning with representations and the development of various representational competence skills (e.g., affordances and limitations, interpret, translate, use).

**Figure 9:**
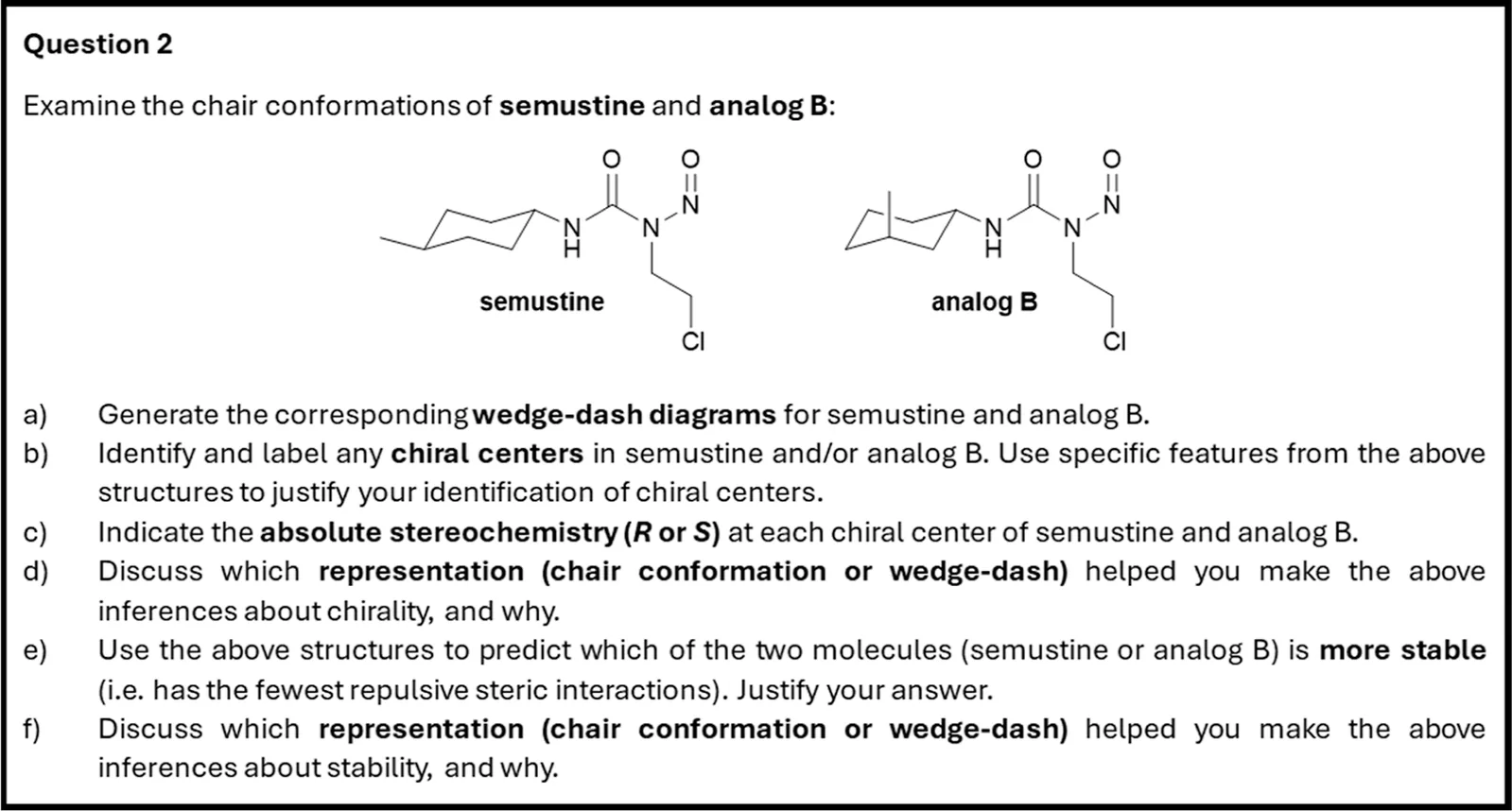
A scaffolded assessment item that explicitly encourages reasoning with representations and the development of various representational competence skills (affordances and limitations, interpret, translate, and use).

## Supplementary Material



## Notes

Information about demographics, interview durations, tables with codes, code definitions, and examples, and supporting figures (PDF)
