# Supplementary material for: Characterizing Student Reasoning with Multiple Representations of Molecular Structure
Source: JACS Au. 2026 Aug 5;6(8):4776–92. doi: 10.1021/jacsau.6c00898 (PMC13508099; doi:10.1021/jacsau.6c00898)

Characterizing Student Reasoning with Multiple Representations of Molecular Structure

Roshni G. Bhaskar[1], Austin Cook[1], Fridah Rotich[1,2], Sara Arévalo[1,3], Maia Popova[1*]

Supporting Information

Contact Information

[1] Department of Chemistry and Biochemistry, University of North Carolina at Greensboro, Greensboro, North Carolina 27412, United States
[2] Center for Instructional Excellence, Purdue University, West Lafayette, Indiana 47907, United States
[3] CNC School of Medicine, University of North Carolina at Chapel Hill, Chapel Hill, North Carolina 27514, United States

Table S1: Codebook of inductively-identified conceptual codes, accompanied by code definitions, and representative quotations

Structural Properties		
**Code**	**Code Definition**	**Representative Quote**
Bond length	References to the length of the bond(s) between two atoms.	I can look at [the structure] automatically and see the difference in the bond length. And I can tell how this portion in this circle and this portion is like flipped. ~Jane
Bond order	References to the number of bonds between two atoms (single-bond, double-bond, etc.)	You see that there's a double bond between the carbon and the nitrogen. That means that they're sharing two pairs of electrons. Otherwise, the other carbons, they are, have single bonds all around them. ~Evans
Bond type	References to the type of chemical bonding between two atoms (covalent, ionic, Van der Waals, etc.)	They wouldn't really do any dipole-dipole moments with any of the other molecules. They'd only do like Van der Waals bonding with other molecules. So, it's intermolecular bonds would be much weaker. ~Victor
Composition	References to the atoms and functional groups present within the provided structure.	There are some carbon-oxygen bonds an alkene, some bromines it has one, two, three, four, five, seven carbons. ~Betty
Connectivity	References to how atoms or functional groups are linked to each other through chemical bonds.	…You can see what's bonded where you can see that the carbon is bonded to the hydrogens, the carbon double bonded to the carbon here. ~Betty
Cyclic System	References to the provided structure being cyclic or acyclic.	It's full of just carbons and hydrogens, so, and it's cyclical. Um, it's going to be the most dissolvable in non-polar solvents, like acetone or hexanes, um. It has only dispersion forces. ~Evans
Hybridization	References to the hybridization (sp^3, sp^2, sp) of a carbon atom in the provided structure.	I can tell that, and I'm looking from left to right here. Uh, carbon one is, um, has sp3 hybridization. I can tell, like, the hybridization of every, um, of every carbon atom. ~Karen
**Spatial Properties**		
**Code**	**Definition**	**Representative Quote**
Bond angle	References to the angle made between two chemical bonds.	In one version you would just see it as a hexagon, but this is kind of giving you a more, a clearer idea of how it's actually shaped because it is still a hexagon from a certain point of view, but it's showing if there are like there's different angles to the bonds, I guess. ~Irene

Bond rotation	References to rotation around a single bond	…and then if we're looking down this bond, all we can see is we're looking at the rotation. So, this, this projection really helps us look at kind of the rotation, not that it's occurring, but in a static form. So, you take a picture of the rotation, right? ~Will
Chirality	References to chirality, chiral centers, or optical rotation.	which if I remember correctly, it's R, but (CHCH3)2 is more important than this one (methyl group in the back carbon in Provided Figure 10), has a higher priority. This CH three (methyl group in the back carbon in Provided Figure 10), has a higher priority than that hydrogen (H in the back carbon in Provided Figure 10) and it, that flows counterclockwise so that one would have an S configuration. ~Nadia
Cis-trans isomerism	Explicit mention of a molecule being cis or trans	So maybe like a trans alkene is more stable than cis, like things like that. ~Betty
Size	General discussion of a molecule being large or small	Every time I see a long chemical formula, I automatically think, is this going to have a high boiling point? Because the bonds are harder to break. I guess it would have a higher melting point as well, for the same reason as it having the high boiling point. ~Jane
Spatial orientation	References to visualizing the orientation of a molecule in three-dimensional space.	Yeah, so I think this does it like a more a good job than the previous ones of like displaying directionality. So, sort of how the bonds are. They're not, not all on 2D now. Some go into the plane; some go out of the plane. ~Charlie
Stereochemistry	References to the existence of stereochemistry, enantiomers, or diastereomers.	So you're following a clockwise rotation, which if I remember correctly, it's R, but (CHCH3)2 is more important than this one (methyl group in the back carbon in Provided Figure 10), has a higher priority. This CH three (methyl group in the back carbon in Provided Figure 10), has a higher priority than that hydrogen (H in the back carbon in Provided Figure 10) and it, that flows counterclockwise so that one would have an S configuration. ~Nadia
Substituent orientation	References to the absolute or relative position of substituents in a molecule (cis or trans substituents, equatorial or axial substituents, etc.).	Bold lines, they're called wedges in stereochemistry and they represent the fact that whatever those wedged lines are connected to they're coming out towards us from the plane of the screen or the paper. ~Evans
Symmetry	Explicit mention of symmetry within a molecule (or lack thereof)	Just because of this OH here, I would say it doesn't have a lot of energy because it's mostly symmetrical except for this OH. ~Vanessa
**Electronic Properties**		
**Code**	**Definition**	**Representative Quote**
Conjugation	References to conjugated systems (alternating single and double bonds).	Um well, we know that there was a hydrogen attached to where the plus sign was and you could make a skeletal structure. You could also see that there's no double bonds, so there's no conjugation. ~Mori
Electron density	References to the distribution of electrons (or the electron cloud) within a molecule.	This would be a polar molecule, meaning that there is electron density or just electron, electronegativity is shifted to one side. ~Nate
Electronegativity	Explicit mention of the concept of electronegativity.	You can tell the electronegativity in a sense…It's going to be very aggressive in trying to bond. ~Nate
Formal charge	Explicit mention of a formal charge or a molecule being positive or negative.	I think the purpose of this representation is to show like the overall formal charge of the compound and to show how many bonds there are. ~Brenda
Inductive effects	Explicit mention of concept; pulling of electron density	Everything will be pulled towards the nitrogen. The nitrogen has a double bond on the left-hand side. That's the strongest of the structure. ~Donna

Intermolecular forces	General discussion of how molecules interact, like hydrogen bonding, or general statement about certain molecules interacting more/less. Mention of Van der Waals, dipole-dipole, or hydrogen bonding.	They'd only do like van der Waals bonding with other molecules. So it's intermolecular bonds would be much weaker. ~Victor
Polarity	General discussion of polar molecules or explicit mention of polarity	I want to say off the top of my head its polar, because of the lone pair electrons group and the removal of this electrons. ~Charlie
Resonance	General discussion of delocalization or explicit mention of resonance or resonance structures	It can change cause it's a resonance structure. ~Vanessa
Valence	General discussion of valence electrons, open electrons, or octet rule	Because it has the four valence electrons so to complete the octet it would need four bonds. ~Irene
**Energetic Properties**		
**Code**	**Definition**	**Representative Quote**
Bond energy	General discussion of bonds breaking and how much energy it might require.	It's a ring, uh, because of the hydroxyl group, I would say that it would require a lot of energy to break off that bond. The molecule in general, uh, would be a strong molecule. ~Charlie
Bond strength	Explicit mention of the strength of a bond.	It communicates that the bonds are singular in between all of the molecules. And you could decide their bond strengths or bond types~ Paula
Diaxial interactions	Discussion surrounding gauche interactions or 1,3 diaxial interactions; or electron cloud overlap.	You have gauche interactions right here (circles the Br and isopropyl group in Drawing 10A). It's pretty bad gauche interaction. ~Will
Energy	General discussion of molecular energy being low, high, stable, unstable, or any other descriptive attribute.	It would be less stable, like higher energy. ~Betty
Potential energy	Resultant discussion surrounding stored chemical energy in bonds, or specific mention of high or low energy states.	So, I think it would be relatively stable. Uh, and yes, uh, energetically, it would be, uh, uh favorable. A low energy state. ~Nate
Ring strain	Explicit mention of ring strain or similar unfavorable cyclic system traits	Like if it's in an energetically favorable conformation, like if it has strain or if it's going to ring or anything it wouldn't really like tell me that. ~Delila
Steric effects	General discussion of steric effects or explicit mention of staggered and eclipsed Newman projections.	So staggered is kind of turned so everything can exist within space and be happy. Versus if they're crammed together in there, it puts, they're going to be meshing with each other, like siblings in the backseat. ~Delila
Stability	General discussion of the tendency of a molecule to resist change due to a variety of factors (such as axial/equatorial, eclipsed/staggered etc.)	I don't think it's going to be that stable because there is like no other methyl groups that are attached to it on the equatorial and other axial position. ~Donna
**Physical Properties**		
**Code**	**Definition**	**Representative Quote**
Acidity and basicity	Discussion surrounding general concepts about Lewis/Bronsted acids and bases and their interactions with electrophilic/nucleophilic atoms	...an acid in the sense that it's going to try and get rid of the hydrogen that's attached to the nitrogen in order to get rid of the positive charge. ~Evans
Hydrophobicity	Specific mention of a structure being hydrophobic due to composition or polarity of substituent groups.	Hydrophobic, I guess, its like a really non-polar atom likely because of all this hydrocarbon it's going to be pretty non-polar I think because of all the hydrocarbons wouldn't like water ~Irene
Molecular weight	Specific mention of how much a molecule weighs as an identifiable physical property.	I know that you can, you can get its molecular structure so you can get its molecular weight. You could weigh it. ~Nate
Melting/boiling point	Inference of a low or high melting/boiling point from other structural properties.	There's going to be hydrogen bonding, which means it's boiling points and melting points will be little bit higher than a normal cyclohexane ~Evans

Reactivity	General discussion surrounding a molecule’s likelihood to react with other molecules due to any variety of molecular properties.	They will probably react with other molecules around it. I think because of the, maybe because of the double bond, like being like, somewhat like highly reactive, maybe. ~Betty
Solubility	General discussion surrounding a molecule’s ability to dissolve in a solvent.	It's going to be soluble in cyclohexanes or hexanes rather than a polar solvent, like water or acetone. ~Evans
**Practical Considerations**		
**Code**	**Definition**	**Representative Quote**
Natural appearance	Use of other molecular properties to infer how a molecule would look in nature.	It's got some conjugation, so it may have a visible color. ~Lena
Cognitive load	Expression of difficulty due to tediousness and a sense of being overwhelmed by the student	So, in a chain like this, the H's are assumed because in certain structures they can be so many, so many hydrogen bonds to represent that it becomes overwhelming. ~Paula
Nomenclature	The use of molecular formula, structure, and/or substituent groups to provide a name for a molecule, or any mention of IUPAC naming system.	So that would mean you could potentially name this molecule based on the presence of a four-carbon chain. It's in a straight line, so that means it's not a ring structure, which means the name would also be different because it's not cyclical ~Paula
Synthetic considerations	Reference to how a molecule was potentially made or synthesized.	What I cannot infer accurately is how the reaction came to be. There’s probably a good amount of ways that this reaction became to be again, I'd like to know how the reaction about how the molecule got its nitrogen as a center. Let's see. Hmm. Well, what else can I not infer, I guess as of right now that's, oh yeah also, how did the carbon lose its uh hydrogen? Yeah, I'd like to also know how it got, how that happened. So, I can't really infer that by looking at those. ~Victor
Simplicity	Expression of increased efficiency and less tediousness when using a particular representation.	It's more efficient, easy to understand a lot also cleaner, not as messy. ~Charlie
Visualization effectiveness	Discussion surrounding a lower cognitive load when viewing a particular representation.	I can actually see the bonds better and how they stack up and connect with one another. ~Charlie

Table S2: Concepts identified as either affordances or limitations, across all six representations. Numbers (X/Y) for a given code imply that the representation was identified as an affordance by X participants and as a limitation by Y participants.

Condensed	Lewis	Skeletal	W-D	Newman	Chairs
**Spatial Properties**					
spatial orientation (0/4)<br>stereochemistry (0/5)	spatial orientation (0/4)	spatial orientation (1/3)	substituent orientation (13/0)<br>stereochemistry (5/0)<br>spatial orientation (4/0)	spatial orientation (4/0)<br>chirality (3/0)	spatial orientation (5/1)<br>substituent orientation (5/0)
**Structural Properties**					
composition (10/1)<br>connectivity (7/7)<br>bond order (6/5)<br>structure (2/4)	bond order (9/0)<br>composition (6/0)<br>connectivity (4/0)<br>structure (4/0)<br>bond type (3/0)	composition (13/2)<br>cyclic system (10/0)<br>bond order (6/0)<br>connectivity (6/0)<br>hybridization (3/0)	composition (10/2)<br>bond order (9/0)<br>connectivity (3/0)	composition (9/0)<br>connectivity (8/2)<br>bond rotation (3/0)	composition (7/1)<br>bond order (6/1)<br>cyclic system (5/0)<br>connectivity (3/0)
**Electronic Properties**					
	formal charge (5/0)<br>electron density (4/0)<br>intermolecular forces (4/0)<br>valence (4/0)<br>polarity (3/0)<br>electronegativity (3/0)	valence (4/0)<br>intermolecular forces (3/0)	electronegativity (4/0)<br>polarity (4/0)<br>intermolecular forces (3/0)		intermolecular forces (3/0)<br>polarity (3/0)
**Physical Properties**					
	mp/bp (3/2)<br>solubility (3/0)	acidity/basicity (4/2)<br>mp/bp (3/0)	mp/bp (2/2)		mp/bp (3/0)
**Energetic Properties**					
energy (1/7)	energy (6/5)<br>stability (3/0)<br>bond strength (3/0)	energy (5/8)<br>stability (4/1)<br>ring strain (3/0)<br>bond energy (3/0)	steric effects (4/0)<br>energy (1/6)	energy (9/2)<br>steric effects (8/0)<br>stability (7/1)	stability (9/1)<br>energy (3/4)
**Practical Considerations**					
simplicity (4/0)		simplicity (12/0)<br>visualization effectiveness (1/4)	visualization effectiveness (4/0)<br>synthetic considerations (3/2)	visualization effectiveness (7/1)	visualization effectiveness (3/0)

affordance	disputed	limitation

Table S3: Heuristics identified in student responses accompanied by definitions and representative quotations

Heuristic	Definition	Representative Quote
Generalization	Pattern recognition and generalization allows the construction of predictions, explanations, and the transfer of knowledge to new situations. However, learned patterns and rules can be overgeneralized, using knowledge about few cases to make hasty conclusions without considering all involved variables. Novice learners in a field are known to overgeneralize learned rules and principles.	*Interviewer:* Cool. And what physical and chemical properties can you not infer from this [condensed] structure?<br>*Evans:* I guess you could like infer solubility again, <u>the less intermolecular forces that are there, the more soluble it's going to be.</u><br><br>*Interviewer:* Any physical or chemical properties [from the condensed structure]?<br>*Ivy:* Probably <u>not going to be water-soluble because it's got a lot of hydrocarbons, I guess.</u> That's all I can come up with from that.
Associative Activation	Associative activation uses linked constructs in the mind to fill in information, quickly and automatically, in situations that resemble past observations or experiences. Past knowledge may be retrieved and used based on irrelevant or superficial similarities, resulting in cognitive biases. Strongly activated information is likely to be given more weight than it deserves, while relevant knowledge that is weakly or not activated will be neglected.	*Interviewer:* All right. Um, what can you infer about chemical and physical properties of the [skeletal structure]?<br>*Jane:* It will be easier, because<u> I know OH is weaker and I always kind of associate OH with like acids and bases</u> and things of that nature.<br><br>*Interviewer:* Every time you see a double bond, do you associate that with an sp2 or is there something more or less, that helps you make sense of that?<br>*Betty:* Well, I mean, I guess, <u>like if I see a pi bond, it's kind of like, oh, well it's probably not sp3</u>, well it's definitely not sp3 hybridized.
One-Reason Decision Making	Humans tend to reduce the number of factors that they analyze when making judgments or constructing explanations, often using the first feature or most prominent feature to provide a plausible answer. The final decision or explanation is based on the variable with the higher cue value. Students' tendency to simplify reasoning by focusing on one single variable and neglecting others is pervasive in chemistry.	*Interviewer:* So, what information can you infer about physical and chemical properties of this [skeletal structure]?<br>*Meghan:* It probably <u>has a high boiling point cause it's big</u>.<br><br>*Interviewer:* So, what information about this molecule can you infer by just looking at this [skeletal structure] here?<br>*Ivy:* Probably <u>not going to be water-soluble because it's got a lot of hydrocarbons</u>, I guess. That's all.
Recognition	When making judgments and decisions, people often rely on information that is easier to retrieve from memory, either because it is computed quickly, or it has been reinforced by frequent exposure. Objects or events that are recognized have a strong influence over the decisions that people make. If one of several objects is recognized and the others are not, students often infer that the recognized object is given a higher value in decision-making or the construction of an explanation.	*Interviewer:* So, how does the double bond and I guess, having a lot of carbons, affect its physical properties?<br>*Irsa:* I don't think it can be right now because there's a double bond in it and I<u> haven't really done any double bond chiral centers. I've only done like single; I've only done alkanes…</u><br><br>*Interviewer:* So, what makes [the condensed structure] not water soluble?<br>*Isabel:* Um, just the fact that it's, um, an alcohol and I guess the best way for me to describe that is <u>when I think of alcohol and water, they don't mix at all.</u> Um, and that's kind of <u>the best way I remember it is by remembering real life instances.</u>

Table S4: Codes referring to the degree of productivity of heuristics, accompanied by code definitions, and representative quotations

Productivity of Heuristics	N/A	Heuristics are seldom used in the construction of claim-supporting explanations. This often co-occurs with the existence of multicomponent reasoning.	*Interviewer*: Does [the Newman Projection] give you any information about the energy of the molecule?<br>*Will*: It gives me information about the stability of the molecule, which I guess in turn could give me information about energy of the molecule. But yeah, it does. So, I know that this is probably not the most stable conformation again, but it's spinning. So, at this, at this point in time, I'm spinning around the axis because I know that it's a, it's a sigma bond, which means its rotation isn't exactly as limited as if it had pi bonding or whatnot.
	Productive	Heuristics are used intentionally and productively, resulting in accurate claims.	*Interviewer*: Yeah. Um, and maybe you can tell me more about [the Newman Projection]. So, what's the difference between staggered and eclipsed?<br>*Delila*: So staggered is kind of turned so everything can exist within space and be happy. Versus if **they're crammed together in there**, it puts, they're going to be meshing with each other**, like siblings in the backseat.** They're just going to fight and they're not as favorable. **They want to be staggered; they want space** (anthropomorphism).
	Semiproductive	Heuristics usage is less intentional, leading to a mixture of accurate, inaccurate and/or oversimplified claims.	*Interviewer*: Okay. And so, what information can you infer about physical and chemical properties from this [Newman Projection]?<br>*Nadia*: So, **I'm not seeing any double bonds, seeing one bromine group. So, it's not going to have high energy**. Like it would if it had double bonds.... [The methyls] are directly in front or behind each other.<br>*Interviewer*: And what does that do to the properties of the molecule?<br>*Nadia*: **It's either going to stabilize it or make it unstable**. It's more of a stable location for the molecules where they are in space. (one-reason decision making, generalization).
	Unproductive	Heuristics are used spuriously and out-of-context, resulting in inaccurate and/or oversimplified claims.	*Interviewer*: Well, I'm just trying to see how this one can be transformed into like a its molecular structure... I'm just curious to know where this OH comes from since I don't see it [in this Newman Projection].<br>*Teresa*: Um, I don't know. **I heard it somewhere about CH becoming OH somehow, but I'm not sure how that worked out**, but I just took a guess. (associative activation, recognition, surface similarity).

Table S5: Codes referring to the accuracy of claims made, accompanied by code definitions, and representative quotations

Accuracy of Claims	Accurate	Participants' claims (and explanations) about the provided structure and its affordances and limitations are accurate.	*Interviewer*: What do the lines represent?<br>*Will*: So, this, **this projection really helps us look at kind of the rotation**, not that it's occurring, but in a static form. So, you take a picture of the rotation, right? Well, why is it keep rotating? Well, because it's going to enter kind of a gauche or the French word for awkward conformation. There's going to be electron repulsion, and that's just going to cause it to keep spinning… But yeah, we're just kind of looking down. It just shows us how to look down the bond. And I don't know, I don't like them as much. **It's easy to determine certain interactions like the gauche interactions or the stability of the molecule**.
	Mostly Accurate	Most of the participants' claims (and explanations) about the provided structure and its affordances and limitations are accurate.	*Interviewer*: Would you say that this [Newman Projection] gives you any information about bonding?<br>*Charlie*: I think the main purpose for me when it comes to Newman is definitely **describing orientation of the molecules**. Um, I think that does affect into like properties of the molecules. Although, I'm having a hard time like explaining and gathering it…<br>*Interviewer*: How about the concept of energy? Do you gather any information about that?<br>*Charlie*: I feel like the orientation, there are possible orientations of the molecule. This is just one orientation of this molecular formula. **And given it can have multiple orientations, some have higher levels of energy than others**.
	Mixed	Participants provide a combination of accurate, inaccurate and/or oversimplified claims (and explanations) about the provided structure and its affordances and limitations.	*Interviewer*: Okay. And so what information can you infer about physical and chemical properties from this [Newman Projection]?<br>*Nadia*: So these overlap each other and these overlap each other in space. So **they are directly in front or behind each other**.<br>*Interviewer*: And what does that do to the properties of the molecule?<br>*Nadia*: It, it's **either going to stabilize it or make it unstable.** It's more of a stable location for the molecules where they are in space.
	Inaccurate	Participants' claims (and explanations) about the provided structure and its affordances and limitations are inaccurate.	*Interviewer*: Okay. So, what do you think this circle [in the Newman Projection] means?<br>*Teresa*: Maybe like how much, I don't, I mean, I feel like maybe like… **Maybe it shows like a quantity of a specific element** or something.<br>*Interviewer*: Okay. So, what information can you infer, um, about this molecule?<br>*Teresa*: Um, it's probably like a, I mean, it's a pie graph, but I don't know much else about it.

Table S6: Number of heuristics identified and productivity of participant reasoning

	Lewis	DW	Chairs	Avg	#Heuristics
Nate	1	1	2	1.33	6
Evans	2	1	2	1.67	3
Charlie	2	2	2	2.00	3
Irene	3	2	1	2.00	5
Paula	2	2	4	2.67	3
Victor	3	2	3	2.67	1
Donna	4	2	3	3.00	8
Ivy	3	3	3	3.00	10
Maggy	3	3	3	3.00	9
Sally	2	3	5	3.33	6
Vanessa	3	4	4	3.67	4
Lorena	4	4	4	4.00	6
Brenda	5	4	4	4.33	5
Mori	5	5	4	4.67	3
Sybil	5	5	4	4.67	2

	Cond	Skeletal	Newman	Avg	#Heuristics
Will	1	1	1	1.00	12
Charlie	2	1	2	1.67	4
Delila	2	2	2	2.00	8
Irsa	2	3	2	2.33	10
Karen	1	4	2	2.33	7
Betty	3	1	3	2.33	8
Lena	2	2	4	2.67	4
Nadia	3	3	3	3.00	8
Meghan	3	2	4	3.00	6
Jane	2	5	3	3.33	6
Isabel	3	4	4	3.67	15
Jorge	3	5	3	3.67	2
Edith	4	5	4	4.33	3
Teresa	5	5	5	5.00	3

Table S7: Codes referring to the relevance of affordances and limitations, accompanied by code definitions, and representative quotations

Relevance of Affordances	Relevant	The affordances described by the participant are relevant to the representation in question and/or align with the conventionally accepted affordances of the representation.	*Interviewer*: What do you think is the purpose of [chair conformations]?<br>*Charlie*: Yeah, so I think this—similar to dash-wedge and Newman—**focuses more on orientation**. I think this might do more of a better job than dash-wedge or Newman [in] **showing 3D on a 2D plane**, it's much more like conceptually [easier] to understand versus I would say a Newman Projection.<br><br>*Interviewer*: And what is the purpose of [chair conformations]?<br>*Nate*: It's to convey the **substituents** on the ring structure **in either, the equatorial or axial positions**… Because if you have substituents in the equatorial, [it's] less favorable, sorry. In the axial, if they're pointing up, then it's going to be less favorable than if they are in the equatorial, which they are now… It shows stability or helps convey stability.
	Sometimes Relevant	The affordances described by the participant are occasionally relevant to the representation in question and/or are inconsistent with the conventionally accepted affordances of the representation	*Interviewer*: What do you think is the purpose of chair conformations?<br>*Lorena*: I think to be able to see it visually. So, you can **identify certain things about the chemical**… whether or not **it's like R or S**… I think that's it.
Relevance of Limitations	Relevant	The limitations described by the participant are relevant to the representation in question and/or align with the conventionally accepted limitations of the representation.	*Interviewer*: Okay. And what, um, information about physical and chemical properties does [Lewis structures] not tell you?<br>*Paula*: Physically, it **doesn't tell me the arrangement of these molecules in space**. So, there'd be different ways that you could draw this or arrange it because it's not, it's not giving me specifics about how it should be represented in a model chemically.
	Sometimes Relevant	The limitations described by the participant are occasionally relevant to the representation in question and/or are inconsistent with the conventionally accepted limitations of the representation.	*Interviewer*: And what does [Lewis structures] not tell you?<br>*Mori*: Um, I guess it wouldn't tell you like about (turns pages in book), it's I don't know, **physical properties, like boiling point** and stuff.

Table S8: Codes referring to students' ability to use structural evidence, accompanied by code definitions, and representative quotations

Use of Structure as Evidence	Very Strong	Participants exhibit an extremely strong ability to use the provided structure as evidence to justify their claims about its affordances and limitations.	*Interviewer*: Okay. Um, what information can you infer about the physical and chemical properties of this structure from this representation?<br>*Karen*: Oh, I can tell that from the structure I'm seeing the **double bond that this is an alkene**. Um, I can tell that, and I'm looking from left to right here. Uh, carbon one is, um, has **sp3 hybridization**. I can tell, like, **the hybridization of every... carbon atom**, um, I can tell also **what's going to be bonded to what, based on its order.** So, the order could tell you, like where everything's going to be bonded to each other. Yeah. That's all I can tell really.
	Strong	Participants exhibit a strong ability to use the provided structure as evidence to justify their claims about its affordances and limitations.	*Interviewer*: Okay. Um, what would you say is the purpose of this representation?<br>*Charlie*: Just purely, just structural. Like it doesn't give you anything else other than the fact that, sorry, it doesn't even give you the structure. **It just shows you the content of the, of the molecule, just to show like what items it has**. Nothing else, really.<br><br>*Interviewer*: Okay. So, what information can you not infer about physical and chemical properties of this structure?<br>*Irsa*: I can't tell if it has, like just from looking at this, I'd have to like figure out myself how many electrons each carbon has or, and like what the actual, like what the structure looks like, **if there is a methyl or not**. Like... **I can infer that from here**, but, um, I also probably would have to draw the, if it's in front or behind the plane myself **and I couldn't tell a boiling point or melting point from this**. Yeah. That's pretty much all I can think of.
	Average	Participants are occasionally able to use the provided structure as evidence to justify their claims about its affordances and limitation. Connections established between claim and evidence are not very strong.	*Interviewer*: So, what information can you infer about physical and chemical properties of this molecule?<br>*Meghan*: Um if I remember correctly, which I probably don't. **It probably has a high boiling point cause it's big**. This is not my strong suit. What else? **A whole bunch of single bonds. I wouldn't say these are double bonds.** I guess it, **I don't remember all the actual properties.**<br><br>*Interviewer*: What information can you infer about the physical or chemical properties from this representation?<br>*Jorge*: **It's an alcohol, because it has that hydroxyl group,** the OH group. It's six carbons long. Yeah. hold on, let me count it again. One, two, three, four. Okay. Yeah, I think it's **six carbons long**.<br>*Interviewer*: So, what would you say is the purpose of this representation?<br>*Jorge*: **The purpose. I'm not entirely sure.**
	Poor	Participants struggle to use the provided structure as evidence to justify their claims about its affordances and limitations.	*Interviewer*: So, what information can you infer about physical and chemical properties of this structure?<br>*Edith*: That it's an **organic bond because it's all carbons, hydrogens, and bromine** which are all organic compounds. Yeah.

Table S9: Codes referring to the presence or absence of multicomponent reasoning, accompanied by code definitions, and representative quotations

Multicomponent Reasoning	Present	Participants successfully connect multiple structural features to multiple concepts, displaying a sophisticated organization of conceptual knowledge and reasoning processes.	*Interviewer*: Okay. So, what information can you infer about physical and chemical properties of this structure?<br>*Nate*: I can infer that this molecule has a **chiral center**, meaning that, what it means is that it has different, uh, different versions of this molecule. So, **if you were to synthesize this molecule**, if this was the product of a reaction you would get, you might get this and you might get another, uh, uh, version as well. Uh, that other version would be, uh, let's see, you have multiple chiral centers, so yeah, you'd have different, uh, molecules and, and they would be, I think the term would be diastereomers, where you essentially, you, you would change each of those positions… If these were to be the products of a reaction, then you might have, uh, this with the Br. Again. So, these don't change, but then you might get this. So now this dash becomes a wedge… You could see that it would be, I guess trying to get the terms **optically active** because you have different, uh, uh, different conformations of this molecule, different, uh, **stereoisomers** um, so it'd be optically active, uh, other chemical properties that I can infer… The other physical properties. I can't really infer anything else in terms of its, you know, boiling point or, or anything to that effect, uh, in terms of its chemical properties, I can say that the, the **bromine is more electronegative** than, than carbon. So, this, it would not be a, a symmetrical, uh, or a, uh, uh, a non-polar molecule. **This would be a polar molecule**, meaning that there is **electron density** or just electron, electronegativity is shifted to one side. So, this side would be more electronegative. So, if it wasn't there, for example, you can draw almost a line down the middle of the molecule and each side would be more or less the same, but because there's a Br there, which is more electronegative, it is not. And I think it would be slightly polar. I don't know how polar would be like, there's a range, but it would be more polar.
	Absent	Participants exhibit relational or single-variable reasoning, connecting a single structural feature to a single concept. Reasoning is often memorization- or heuristics-based.	*Interviewer*: That's fine. Okay. So, what information about physical and chemical properties of this structure, can you infer from this representation?<br>*Sally*: Well, **it has a full octet I guess because there are no formal charges**, I guess. So, I have no idea about like, so I know the properties we typically look at are like the dipole moment. So, I guess I could say that for this one, because **I'm going to assume that Bromine has a partial negative, which makes the carbon a partial positive charge**. I'm gonna assume that it has a dipole moment that goes to this point. I don't know the electronegativity of bromine, but because of that, I guess I could say that… I don't know.<br><br>*Interviewer*: Okay. So, what information can you infer about physical and chemical properties of this structure?<br>*Vanessa*: Well, I can infer that… **I don't think it would be an acid because the OH, so it would probably be a base**… And then, I don't know about physically.

Table S10: Reasoning tiers and their corresponding characteristics identified across all representations

Tier	Attributes	Condensed Structures	Lewis Structures	Skeletal Structurers	Wedge-Dash Diagrams	Newman Projections	Chair Conformations
1	Number (and relevance) of affordances	Few-many, relevant affordances	Many, relevant affordances	Many, relevant affordances	Many, relevant affordances	Many, relevant affordances	Many relevant affordances
	Number (and relevance) of limitations	Many, relevant limitations	Many, relevant limitations	Few, relevant limitations	Few, relevant limitations	N/A	Few relevant limitations
	Accuracy of claims	Accurate	Accurate	Accurate	Accurate	Accurate	Accurate
	Use of structure as claim-supporting evidence	Always	Always	Always	Always	Always	Always
	Multicomponent Reasoning	Yes	Yes	Yes	Yes	Yes	Yes
	Frequency of heuristics use	Infrequent-to-none	Infrequent	Frequent	None	None	None
	Productivity of heuristics use	Productive	Productive	Productive	NA	NA	NA
2	Number (and relevance) of affordances	Few-to-many, relevant affordances	Many, relevant affordances	Many, relevant affordances	Many, relevant affordances	Many, relevant affordances	Many relevant affordances
	Number (and relevance) of limitations	Few-to-many, relevant limitations	Few, relevant limitations	Few-many, relevant Ls	Few, relevant limitations	Few, semi-relevant limitations	Few relevant limitations
	Accuracy of claims	Accurate	Accurate	Mostly accurate	Accurate	Mostly accurate	Accurate
	Use of structure as claim-supporting evidence	Almost always	Almost always	Always	Always	Always	Sometimes
	Multicomponent Reasoning	No	No	No	No	No	No
	Frequency of heuristics use	Frequent	Frequent	Frequent	Infrequent	Infrequent	Frequent
	Productivity of heuristics use	Productive	Productive	Semiproductive	Productive	Productive	Productive
3	Number (and relevance) of affordances	Few-to-many, sometimes relevant affordances	Many, relevant affordances	Many, relevant affordances	Few, relevant affordances	Few-to-many relevant affordances	Few relevant affordances
	Number (and relevance) of limitations	Few, relevant limitations	Few, sometimes relevant limitations	Few, relevant limitations	Few, sometimes relevant limitations	Few-to-no semi-relevant limitations	Few relevant limitations
	Accuracy of claims	Mixed	Mixed	Mixed	Mixed	Mixed	Mixed
	Use of structure as claim-supporting evidence	Sometimes	Almost always	Sometimes	Sometimes	Sometimes	Sometimes
	Multicomponent reasoning	No	No	No	No	No	No
	Frequency of heuristics use	Frequent	Frequent	Frequent	Frequent	Frequent	Frequent
	Productivity of heuristics use	Semi-productive	Semi-productive	Semi-productive	Semiproductive	Semi-productive	Semiproductive
4	Number (and relevance) of affordances	Few-to-many, sometimes relevant affordances	Few, relevant affordances	Many, relevant affordances	Few, relevant affordances	Few-to-many relevant affordances	Few sometimes relevant affordances
	Number (and relevance) of limitations	Few, relevant limitations	Few, sometimes relevant limitations	Few, sometimes relevant limitations	Few, sometimes relevant limitations	Few-to-no semi-relevant limitations	Few relevant limitations
	Accuracy of claims	Mixed	Inaccurate	Mixed	Mixed	Mixed	Inaccurate
	Use of structure as claim-supporting evidence	No	No	No	Sometimes	No	Sometimes
	Multicomponent reasoning	No	No	No	No	No	No
	Frequency of heuristics use	Frequent	Frequent	Frequent	Frequent	Frequent	Frequent
	Productivity of heuristics use	Semi-productive	Unproductive	Unproductive	Unproductive	Semi-productive	Unproductive
5	Number (and relevance) of affordances	Few, sometimes relevant affordances	Few, relevant affordances	Few, sometimes relevant affordances	Few, relevant affordances	No relevant affordances	Few sometimes relevant affordances
	Number (and relevance) of limitations	Few, sometimes relevant limitations	Few, sometimes relevant limitations	None-few, sometimes relevant limitations	No relevant limitations	No relevant limitations	No relevant limitations
	Accuracy of claims	inaccurate/NA	NA	Inaccurate	Mixed	Inaccurate	Inaccurate
	Use of structure as claim-supporting evidence	No	No	No	Sometimes	No	No
	Multicomponent reasoning	No	No	No	No	No	No
	Frequency of heuristics use	NA	NA	Frequent	NA	Frequent	NA
	Productivity of heuristics use	NA	NA	Unproductive	NA	Unproductive	NA

Table S11: Summary of Demographic Information

**Major**	Biology (54%)	Chemistry and Biochemistry (25%)		Other (21%)
**Gender**	Female (79%)		Male (21%)	
**Ethnicity**	Black/African American (36%)	White/Caucasian (32%)	Asian/Pacific Islander (14%)	Other (18%)

Our sample included majors in biology (54%) and chemistry and biochemistry (25%). Most (79%) participants identified as female. The sample was ethnically diverse (36% Black/African American, 32% White/Caucasian, 14% Asian/Pacific Islander, 18% other ethnicities) and represents the population of the course and the institution from which participants were recruited.

Figure S1. The number of affordances (positive y-axis) and the number of limitations (negative y-axis) identified by participants in reasoning tiers 1 (top), 3 (middle) and 5 (bottom). Participants identify fewer affordances and limitations across fewer conceptual categories as we move from reasoning tiers 1 through 5.

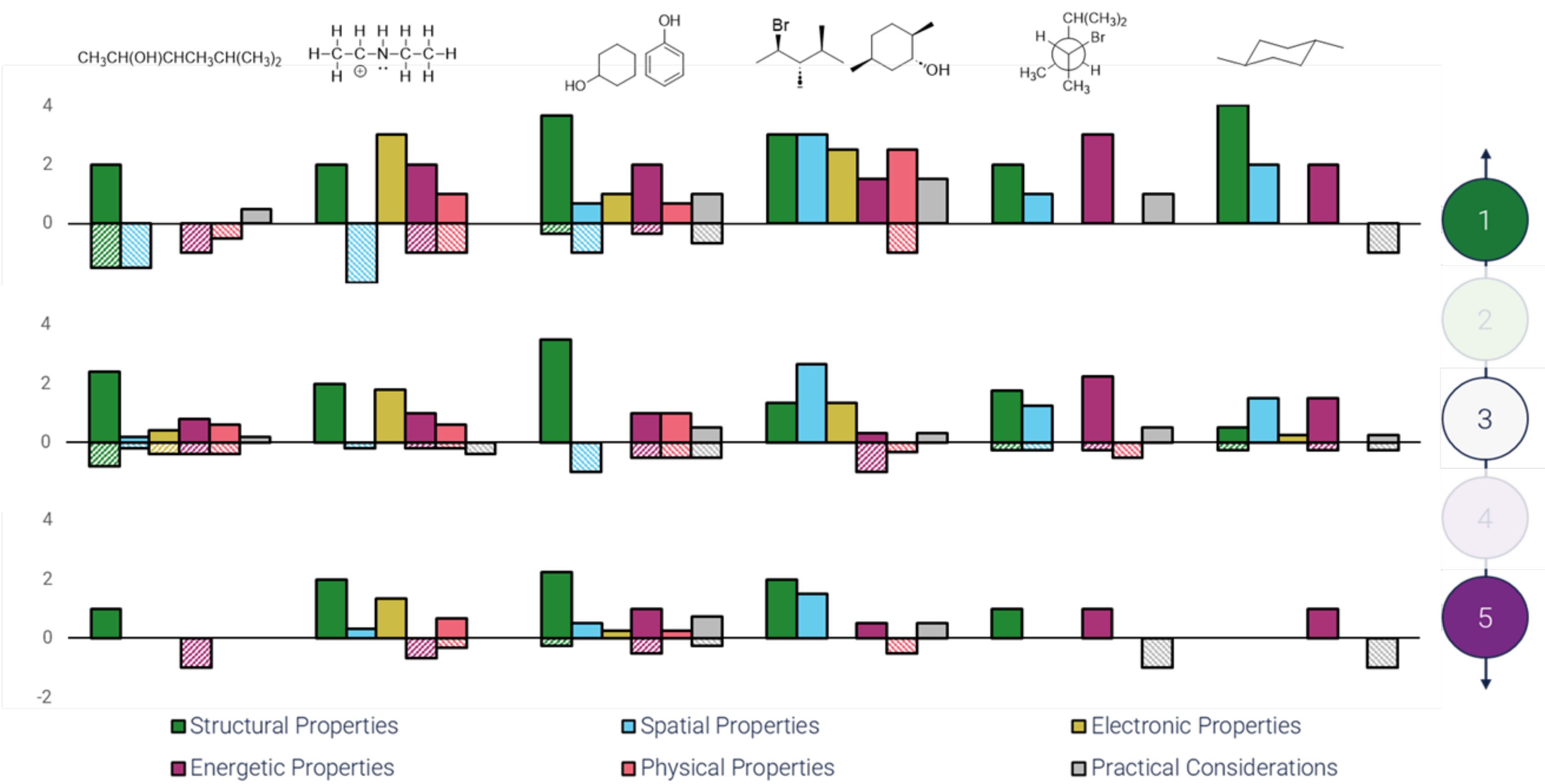

Supplement: Supplementary file 1 [file au6c00898_si_001.pdf]
